# The Limits and Intensity of *Plasmodium* *falciparum* Transmission: Implications for Malaria Control and Elimination Worldwide 

**DOI:** 10.1371/journal.pmed.0050038

**Published:** 2008-02-26

**Authors:** Carlos A Guerra, Priscilla W Gikandi, Andrew J Tatem, Abdisalan M Noor, Dave L Smith, Simon I Hay, Robert W Snow

**Affiliations:** 1 Malaria Public Health and Epidemiology Group, Centre for Geographic Medicine, Kenyan Medical Research Institute–University of Oxford–Wellcome Trust Collaborative Programme, Nairobi, Kenya; 2 Spatial Ecology and Epidemiology Group, Department of Zoology, University of Oxford, Oxford, United Kingdom; 3 Centre for Tropical Medicine, John Radcliffe Hospital, University of Oxford, Oxford, United Kingdom; 4 Department of Zoology and Emerging Pathogens Institute, University of Florida, Gainesville, Florida, United States of America; Royal Melbourne Hospital, Australia

## Abstract

**Background:**

The efficient allocation of financial resources for malaria control using appropriate combinations of interventions requires accurate information on the geographic distribution of malaria risk. An evidence-based description of the global range of Plasmodium falciparum malaria and its endemicity has not been assembled in almost 40 y. This paper aims to define the global geographic distribution of P. falciparum malaria in 2007 and to provide a preliminary description of its transmission intensity within this range.

**Methods and Findings:**

The global spatial distribution of P. falciparum malaria was generated using nationally reported case-incidence data, medical intelligence, and biological rules of transmission exclusion, using temperature and aridity limits informed by the bionomics of dominant *Anopheles* vector species. A total of 4,278 spatially unique cross-sectional survey estimates of P. falciparum parasite rates were assembled. Extractions from a population surface showed that 2.37 billion people lived in areas at any risk of P. falciparum transmission in 2007. Globally, almost 1 billion people lived under unstable, or extremely low, malaria risk. Almost all P. falciparum parasite rates above 50% were reported in Africa in a latitude band consistent with the distribution of Anopheles gambiae s.s. Conditions of low parasite prevalence were also common in Africa, however. Outside of Africa, P. falciparum malaria prevalence is largely hypoendemic (less than 10%), with the median below 5% in the areas surveyed.

**Conclusions:**

This new map is a plausible representation of the current extent of P. falciparum risk and the most contemporary summary of the population at risk of P. falciparum malaria within these limits. For 1 billion people at risk of unstable malaria transmission, elimination is epidemiologically feasible, and large areas of Africa are more amenable to control than appreciated previously. The release of this information in the public domain will help focus future resources for P. falciparum malaria control and elimination.

## Abstract 2

Combining extensive surveillance and climate data, as well as biological characteristics of *Anopheles* mosquitoes, Robert Snow and colleagues create a global map of risk for *P. falciparum* malaria.

## Editors' Summary

**Background.:**

Malaria is a parasitic disease that occurs in tropical and subtropical regions of the world. 500 million cases of malaria occur every year, and one million people, mostly children living in sub-Saharan Africa, die as a result. The parasite mainly responsible for these deaths—Plasmodium falciparum—is transmitted to people through the bites of infected mosquitoes. These insects inject a life stage of the parasite called sporozoites, which invade and reproduce in human liver cells. After a few days, the liver cells release merozoites (another life stage of the parasite), which invade red blood cells. Here, they multiply before bursting out and infecting more red blood cells, causing fever and damaging vital organs. Infected red blood cells also release gametocytes, which infect mosquitoes when they take a human blood meal. In the mosquito, the gametocytes multiply and develop into sporozoites, thus completing the parasite's life cycle. Malaria can be treated with antimalarial drugs and can be prevented by controlling the mosquitoes that spread the parasite (for example, by using insecticides) and by avoiding mosquito bites (for example, by sleeping under a insecticide-treated bednet).

**Why Was This Study Done?:**

Because malaria poses such a large global public-health burden, many national and international agencies give countries where malaria is endemic (always present) financial resources for malaria control and, where feasible, elimination. The efficient allocation of these resources requires accurate information on the geographical distribution of malaria risk, but it has been 40 years since a map of malaria risk was assembled. In this study, which is part of the Malaria Atlas Project, the researchers have generated a new global map to show where the risk of P. falciparum transmission is moderate or high (stable transmission areas where malaria is endemic) and areas where the risk of transmission is low (unstable transmission areas where sporadic outbreaks of malaria occur).

**What Did the Researchers Do and Find?:**

To construct their map of P. falciparum risk, the researchers collected nationally reported data on malaria cases each year and on the number of people infected in sampled communities. They also collected information about climatic conditions that affect the parasite's life cycle and consequently the likelihood of active transmission. For example, below a certain temperature, infected mosquitoes reach the end of their natural life span before the parasite has had time to turn into infectious sporozoites, which means that malaria transmission does not occur. By combining these different pieces of information with global population data, the researchers calculated that 2.37 billion people (about 35% of the world's population) live in areas where there is some risk of P. falciparum transmission, and that about 1 billion of these people live where there is a low but still present risk of malaria transmission. Furthermore, nearly all the regions where more than half of children carry P. falciparum parasites (a P. falciparum prevalence of more than 50%) are in Africa, although there are some African regions where few people are infected with P. falciparum. Outside Africa, the P. falciparum prevalence is generally below 5%.

**What Do These Findings Mean?:**

The accuracy of this new map of the spatial distribution of P. falciparum malaria risk depends on the assumptions made in its assembly and the accuracy of the data fed into it. Nevertheless, by providing a contemporary indication of global patterns of P. falciparum malaria risk, this new map should be a valuable resource for agencies that are trying to control and eliminate malaria. (A similar map for the more common but less deadly P. vivax malaria would also be useful, but has not yet been constructed because less information is available and its biology is more complex.) Importantly, the map provides an estimate of the number of people who are living in areas where malaria transmission is low, areas where it should, in princple, be possible to use existing interventions to eliminate the parasite. In addition, it identifies large regions of Africa where the parasite might be more amenable to control and, ultimately, elimination than previously thought. Finally, with regular updates, this map will make it possible to monitor the progress of malaria control and elimination efforts.

**Additional Information.:**

Please access these Web sites via the online version of this summary at http://dx.doi.org/10.1371/journal.pmed.0050038.

- The MedlinePlus encyclopedia contains a page on malaria (in English and Spanish)
- Information is available from the World Health Organization on malaria (in English, Spanish, French, Russian, Arabic, and Chinese)
- The US Centers for Disease Control and Prevention provide information on malaria (in English and Spanish)
- Information is available from the Roll Back Malaria Partnership on its approach to the global control of malaria
- More information is available on global mapping of malaria risk from the Malaria Atlas Project

## Introduction

The magnitude of the public health burden posed by malaria worldwide [1] and its connection to poverty [2] has galvanized the international donor community to put malaria control high on the development agenda and helped leverage unprecedented additional financing for malaria endemic countries [3]. Progress toward agreed targets of intervention coverage has been slow [4–6], but recent evidence indicates a precipitous increase in access to effective drugs and prevention strategies in several countries [7–10]. In part, this renaissance in malaria control has served as a catalyst to revisit the possibility of malaria elimination in many regions and countries [11–14]. A changing malaria landscape requires an accurate spatial and dynamic description of malaria risk that maps the spatial extent and need for control and elimination over the coming decades. Such a map is conspicuous by its absence [15].

Here, we present the first detailed description of the global distribution of P. falciparum risk in 40 y [16,17] by using geopositioned assemblies of national surveillance of malaria risk, medical intelligence, biological models of transmission suitability, and surveys of parasite prevalence. The paper focuses on detailing the data sources and their adaptation for the malaria cartography necessary to guide current disease control, with an emphasis on how we define the spatial limits of stable and unstable P. falciparum risk worldwide.

## Methods

### Using Medical Intelligence to Define the Limits of P. falciparum Risk

Many countries have information assembled from medical intelligence on the distribution of malaria risk within their national borders. This information is documented primarily in reports from national health information systems that define the annual numbers of confirmed parasite-specific local malaria infections by geographic unit, referred to classically as the annual parasite incidence (API) [18–21]. The API is generated from various combinations of active (fever surveys in communities where every person presenting with a fever is tested for parasite infection) and passive (reports from febrile patients attending the local health services) case detection, and usually expresses the combined results as the number infected per 1,000 people per annum (pa) [18–21]. The precision of these estimates of malaria incidence are highly variable, and with the exception of some countries where case identification is a primary control tool [22], these data cannot be used confidently to derive the public health burden posed by malaria [1,23–26]. They can, however, be a useful indicator of where local parasite species-specific malaria risk is likely or absent, and are particularly plausible when triangulated with other sources of medical intelligence, reported in international travel health guidelines or by national malaria control programmes.

Malaria coordinating officers in the regional offices of the World Health Organization (WHO), responsible for the collation of national API data from member countries were contacted to obtain data reported nationally to the highest possible geographic administrative unit level on populations at risk and numbers of confirmed P. falciparum cases, for as many years as were available between 2002 and 2006. Among the countries in the American Regional Office, P. falciparum–specific API (*Pf*API) data from national surveillance systems in Brazil, Colombia, Peru, and Honduras were obtained directly from personal communication with malaria specialists. The reported cases of confirmed P. falciparum malaria per 1,000 resident population were computed for each year by administrative level and averaged over the number of reporting years. Summary data were categorized as no autochthonous P. falciparum cases reported, <0.1 autochthonous P. falciparum cases per 1,000 people pa, and ≥0.1 autochthonous P. falciparum cases per 1,000 people pa. The threshold around 0.1 cases per thousand pa was used to provide some indication of unstable conditions versus more stable transmission. This threshold is consistent with previous uses of *Pf*API during the Global Malaria Eradication Programme [27] and balanced against the confidence in the precision of reported *Pf*API values (Protocol S1). Each *Pf*API summary estimate was mapped by matching it to its corresponding first-, second-, or third-level administrative unit in a geographic information system (GIS; ArcView GIS 3.2, ESRI, 1999).

Mapped *Pf*API data were then compared to other sources of medical intelligence, notably national malaria control presentations at regional malaria meetings obtained from regional WHO malaria coordinators and from Web sites, published sources that described national malaria epidemiology, and international travel and health guidelines [28,29]. These combined approaches were particularly useful to identify mapped descriptions of risk defined at higher spatial resolution than those described by the *Pf*API reported across large first-level administrative units. Details of all sources used are provided in Protocol S1.

### Defining the Biological Limits of P. falciparum Transmission

Within the limits of risk described through *Pf*API, environmental conditions suitable for transmission vary enormously. These variations can be captured at much higher spatial resolution than it is possible to define by stratifying risk at administrative unit levels. Climate-based determinants of parasite and vector development and survival were developed that impose biological constraints on the geographical limits of P. falciparum transmission.

First, we used a combination of the temperature-dependant relationship between P. falciparum sporogony and the longevity of the main dominant vectors to estimate the proportion of vectors surviving parasite development (Protocol S2). Using mean monthly temperature records from a 30-arcsec (∼1 km) spatial resolution climate surface [30], the duration of P. falciparum sporogony was estimated for each synoptic calendar month, and those pixels where the duration of sporogony was 31 d or less were identified. The exception was small areas that potentially support the longer-lived Anopheles sergentii and A. superpictus, where 62 d were considered more appropriate biologically (Protocol S2). This resulted in 12 images with a binary outcome: P. falciparum sporogony could or could not be completed in the month. These images were then combined to identify the number of suitable months for P. falciparum transmission in a synoptic year. All pixels where the duration of sporogony exceeded 1 mo, or 2 mo for areas within the range of A. sergentii and A. superpictus, were masked since it was highly unlikely that transmission would occur.

Second, there are areas within several malaria endemic countries where, despite temperature being suitable for sporogony, arid conditions restrict *Anopheles* development and survival [31]. Limited surface water reduces the availability of water bodies for oviposition. Moreover, low ambient humidity in arid environments further affects egg and adult survival through the process of desiccation [32]. The ability of adult vectors to survive long enough to contribute to parasite transmission and of preadult stages to ensure minimum population abundance is, therefore, dependent on the levels of aridity and species-specific resilience to arid conditions. To capture the influence of aridity on transmission we used the enhanced vegetation index (EVI) derived from the bidirectional reflectance-corrected MODerate-resolution Imaging Spectroradiometer (MODIS) sensor imagery, available at approximately 1-km spatial resolution [33,34] (Protocol S2). Temporal Fourier–processed, monthly EVI images were used to develop 12 monthly surfaces that reclassified EVI ≤ 0.1, assuming this corresponded to a good proxy for arid conditions [35,36]. Pixels were classified as suitable for transmission if their EVI values were higher than 0.1 for at least two consecutive months in an average year. This definition was based on the biological requirement, at optimum temperatures, of at least 12 d to complete vector development from egg to adult [37] and on the assumption that a second month is required for a sufficient vector population to establish and transmit malaria [38]. These reclassified aridity images were then overlaid in a GIS to produce 12 paired images. The 12 pairs were then combined to define pixels where conditions were suitable for transmission. The aridity mask was treated differently from the temperature-limiting mask to allow for the possibility, in arid environments, of highly over-dispersed transmission due to man-made water collection points and nomadic human populations transporting vectors and parasites [39–41]. A more conservative approach was taken, therefore, which down-regulated *Pf*API risk by one class. In other words, extremely arid areas defined originally as at stable risk were stepped down to unstable risk and those classified initially as unstable to malaria free.

### Estimating Populations at P. falciparum Transmission Risk in 2007

The Global Rural Urban Mapping Project alpha version provides gridded population counts and population density estimates for the years 1990, 1995, and 2000, both adjusted and unadjusted to the United Nations' national population estimates [42]. We used the adjusted population counts for the year 2000 and projected them to 2007 by applying national, medium variant, intercensal growth rates by country [43], using methods previously described [44]. This resulted in a contemporary population density surface of approximately 1-km spatial resolution, which was combined with the climate-adjusted *Pf*API risk surface to extract population at risk estimates using ArcView GIS 3.2 (ESRI, 1999).

### Describing Global Patterns of Parasite Prevalence

We have described previously the rigorous process of identifying, assembling, and geolocating community-based survey estimates of parasite prevalence undertaken since 1985 [45]. These data were used here to define the ranges of P. falciparum parasite prevalence rates (*Pf*PR) in areas of stable and unstable malaria risk by WHO region. We acknowledge that these geopolitical boundaries do not necessarily conform to ecological or biological spatial representations of malaria [46,47]. They do, however, represent coherent regions of collective planning and cooperation for malaria control. In an attempt to minimize epidemiologically unrealistic divides for summary purposes, we have combined the Southeast Asian (SEARO) and Western Pacific (WPRO), as well as the Eastern Mediterranean (EMRO) and European (EURO) regions. The American WHO region (AMRO) and the African WHO region (AFRO) were considered separately. *Pf*PR estimates were reported in various age groupings. To standardize to a single, representative age range of 2–10 y, we applied an algorithm based on catalytic conversion models first adapted for malaria by Pull and Grab [48] and described in detail elsewhere [49]. The geolocated and age-standardized prevalence data (*Pf*PR_2−10_) [45] were overlaid on the *Pf*API risk surface to extract a corresponding *Pf*API value.

## Results

### *Pf*API Data and Medical Intelligence to Define Spatial Limits of Transmission

**Figure 1:**
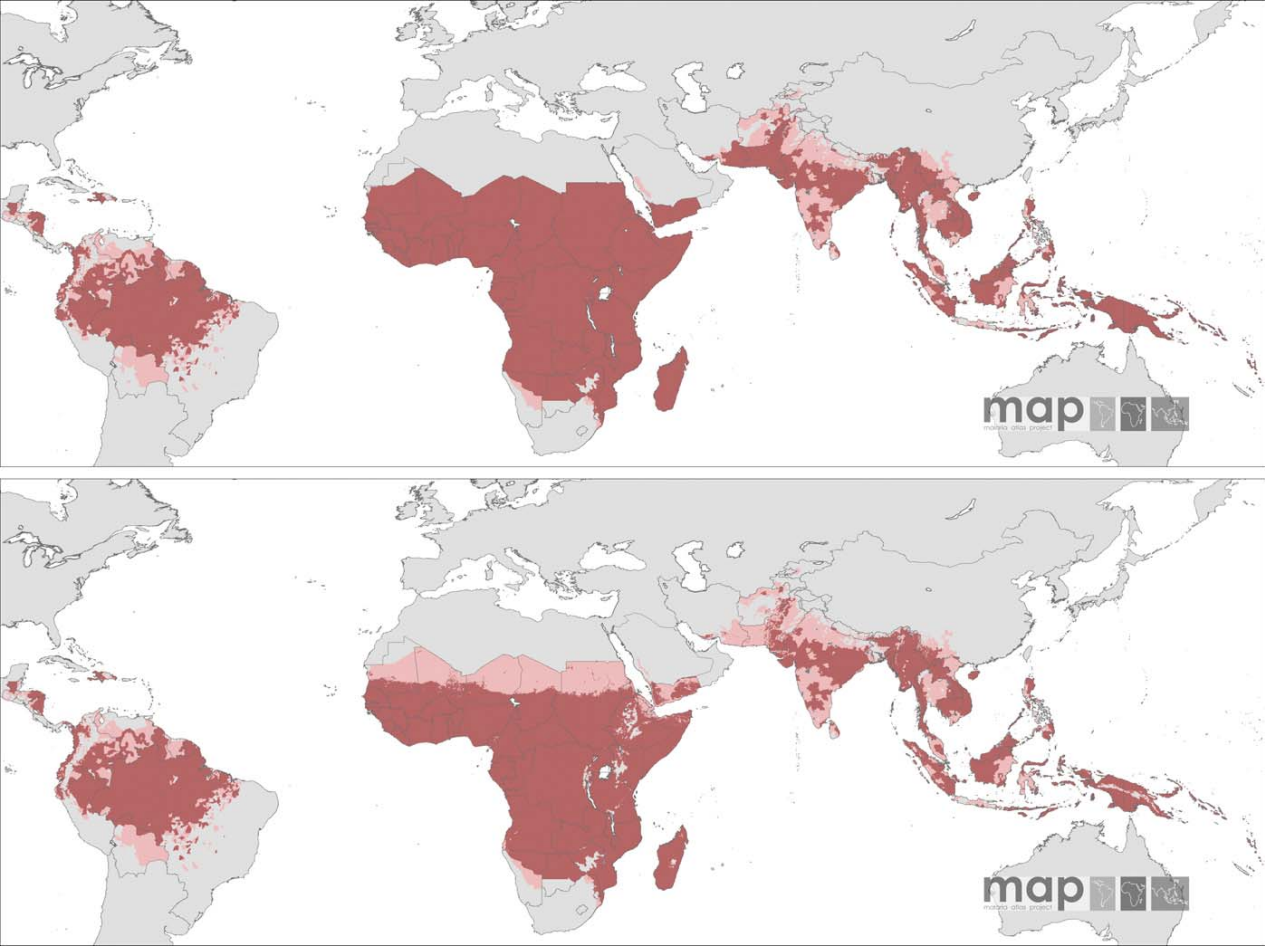
P. falciparum Malaria Risk Defined by Annual Parasite Incidence (top), Temperature, and Aridity (bottom) Areas were defined as stable (dark-red areas, where *Pf*API ≥ 0.1 per thousand pa), unstable (pink areas, where *Pf*API < 0.1 per thousand pa), or no risk (light grey). The few areas for which no *Pf*API data could be obtained, mainly found in India, are coloured in dark grey. The borders of the 87 countries defined as P. falciparum endemic are shown. Highland areas where risk was excluded due to temperature appear in light grey. The aridity mask excluded risk in a step-wise fashion, reflected mainly in the larger extents of unstable (pink) areas compared to the top panel, particularly in the Sahel and southwest Asia (southern Iran and Pakistan).

The *Pf*API data identified 87 countries at risk of P. falciparum transmission between 2002 and 2006, which we now consider as P. falciparum endemic countries (*Pf*MEC) in 2007 (Protocol S1). *Pf*API data were mapped to first, second, or third administrative level units across 41 *Pf*MECs covering a total of 8,789 unique polygons. These data incorporate complete years between 2002 and 2006, including summaries of three consecutive years for 16 countries, two consecutive years for eight countries, and the most recent complete year for 17 countries (Protocol S1). No information was available for 46 countries; mostly those in Africa. The spatial representation of no risk, unstable (*Pf*API < 0.1 per 1,000 people pa), and stable risk (*Pf*API ≥ 0.1 per 1,000 people pa) of P. falciparum transmission globally is shown in Figure 1, top panel.

### Temperature and Aridity Masks to Constrain Limits of Transmission

Within the *Pf*API limits of stable transmission (*Pf*API ≥ 0.1 per 1,000 pa) on the African continent, the areas with no temperature-suitable months for transmission were congruent with the high altitude areas in Ethiopia, Eritrea, western Kenya, eastern Tanzania, Rwanda, Burundi, eastern Democratic Republic of the Congo, the Malagasy highlands, Mount Cameroon, and the eastern highland ranges in Zimbabwe (Figure 1, bottom panel). Outside of Africa, there was a close correspondence between the areas masked by the absence of reported autochthonous cases and areas classified as unsuitable for transmission based on low temperature in Andean and Himalayan areas (Figure 1, bottom panel). The application of the temperature mask provided a finer spatial resolution constraint to *Pf*API data, particularly for the island of New Guinea and the highlands neighbouring the city of Sana'a, Yemen. Important reductions in the spatial areas of risk were also evident in some administrative units in Afghanistan, Bhutan, China, India, and Kyrgyzstan.

The aridity mask constrained the mapped P. falciparum transmission risk to small pockets in large administrative boundaries from southern areas of Hilmand and Kandahar, in Afghanistan, the municipality of Djibouti, in Djibouti, and the south-eastern provinces of Iran. The risk areas along the Red Sea coast of Saudi Arabia were also reduced further using the aridity mask. Additional areas constrained within their spatial margins to no risk using the aridity mask included administrative units in India (*n* = 4), Pakistan (*n* = 9), Peru (*n* = 3), Kyrgyzstan (*n* = 2), Tajikistan (*n* = 1), and the low risk areas of Namibia bordering the Namib desert. Large areas covered by the aridity mask were reduced from stable (*Pf*API ≥ 0.1 per 1,000 pa) to unstable risk (*Pf*API < 0.1 per 1,000 pa) in the Sahel. The transmission reducing effects of aridity were also evidenced in Djibouti, Eritrea, northwest Kenya, northeast Ethiopia, northern Somalia, central and coastal areas of Yemen, and southern Pakistan. Importantly, these areas retained small pockets of higher, more-suitable transmission conditions, corresponding to river tributaries and irrigated land where higher transmission risk is supported [50].

### Populations at Risk

Table 1 provides a summary of the spatial extents and the projected 2007 populations at risk (PAR) within areas of assumed unstable (*Pf*API < 0.1 per 1,000 pa) and stable P. falciparum transmission (*Pf*API ≥ 0.1 per 1,000 pa) globally and by WHO region. Country PAR estimations are also provided (Table S1). We estimate that there are 2.37 billion people at risk of P. falciparum transmission worldwide, 26% located in the AFRO region and 62% in the combined SEARO-WPRO regions (Table 1). The definition of unstable risk outlined here is the predominant feature of exposure to transmission in the EMRO-EURO region (Table 1). Low-risk areas in AFRO were also coincident with arid, low population density areas. Globally, 42% of the population exposed to some risk of P. falciparum was classified as inhabiting areas of unstable transmission; the total population in these areas was 0.98 billion people.

**Table 1:**
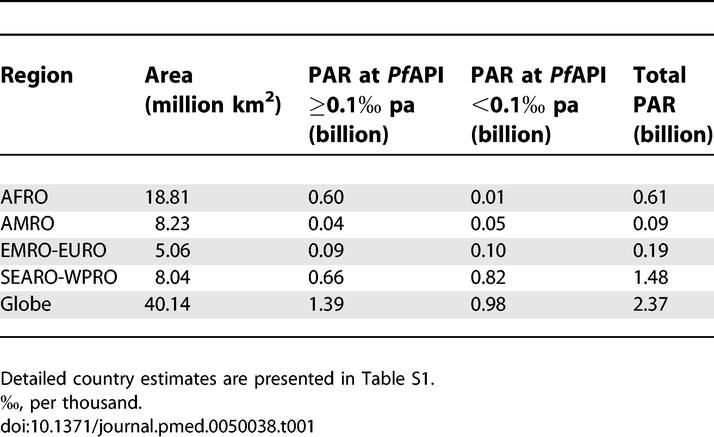
Area and Population at Risk of P. falciparum Malaria in 2007

### Global and Regional Summary of P. falciparum Parasite Prevalence

The summary data on age-corrected *Pf*PR are presented without adjustments for biological and climatic covariates, urbanization, congruence with dominant *Anopheles* vector species, or any sampling issues inherent in an opportunistic sample of this kind. This is the subject of ongoing work. The summarized data, however, do provide important new insights into the ranges of infection prevalence reported between regions of the world within the P. falciparum spatial limits of stable and unstable transmission. A total of 4,278 spatially unique cross-sectional survey estimates of *Pf*PR were assembled as part of the activities of the Malaria Atlas Project (MAP) by 01 September 2007. These included 186 (4.4%) surveys that were not possible to geolocate and are not considered further in the analysis. Of the positioned survey data, 3,700 (90.4%) were derived from individual communities (about 10 km^2^ or less), 131 from wide areas (more than about 10 km^2^ and about 25 km^2^ or less), 145 from small polygons (more than about 25 km^2^ and about 100 km^2^ or less), and 116 from large polygons (more than about 100 km^2^) [45]. A total of 406 surveys were undertaken outside the defined spatial limits of P. falciparum transmission, of which 46 reported presence of P. falciparum infection in the populations surveyed and 360 reported zero prevalence after allowing for a 10-km buffer around the limits. Thus, the overall sensitivity adjusting for plausible positioning errors [51] was 98.5%. There were 611 surveys falling inside the limits that reported zero prevalence. Even using the 10-km buffer the specificity of the limits was low (37.1%). This reflects the difficulties in estimating zero prevalence without large sample sizes [52], as well as the over-dispersed nature of infection risks between communities within small spatial scales [53].

**Figure 2:**
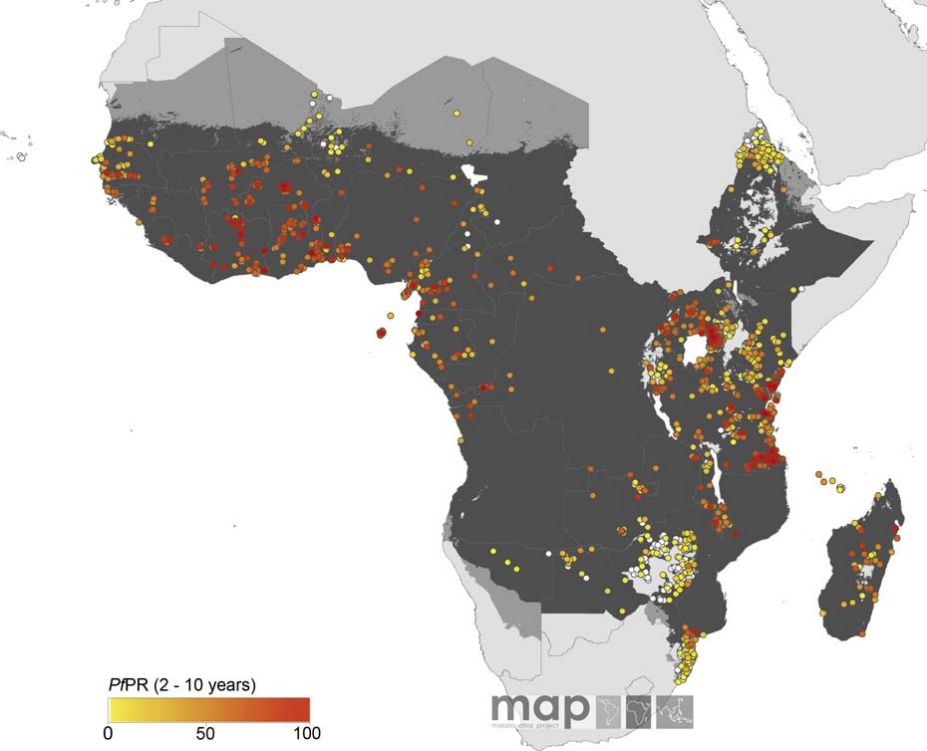
Community Surveys of P. falciparum Prevalence Conducted between 1985 and 2007 in AFRO Other regions are shown in Figures 3–5. Of the 4,278 surveys reported globally, 4,092 could be geopositioned of which 3,686, shown in these figures, fell within the predicted limits of P. falciparum malaria risk. A total of 406 records, not shown in the figures, were found outside the limits, of which 46 reported presence of P. falciparum. Data shown are age-standardized (*Pf*PR_2–10_) and represented as a continuum from zero to 100%. Table 2 and Figure 6 present detailed descriptive statistics for these data.

**Figure 3:**
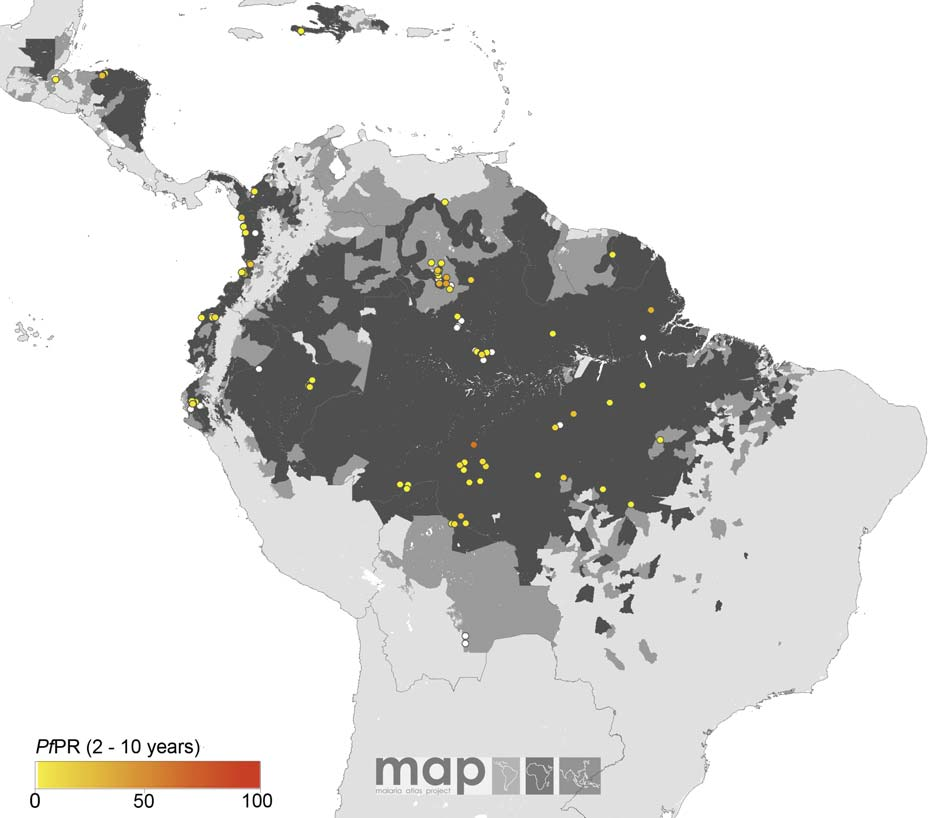
Community Surveys of P. falciparum Prevalence Conducted between 1985 and 2007 in AMRO

**Figure 4:**
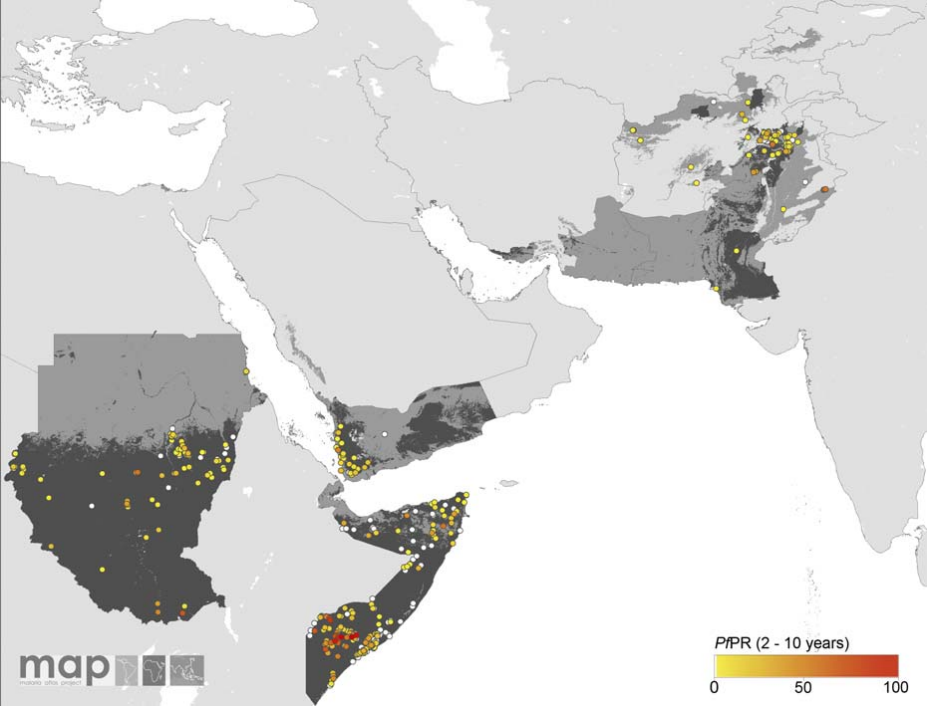
Community Surveys of P. falciparum Prevalence Conducted between 1985 and 2007 in EMRO-EURO

**Figure 5:**
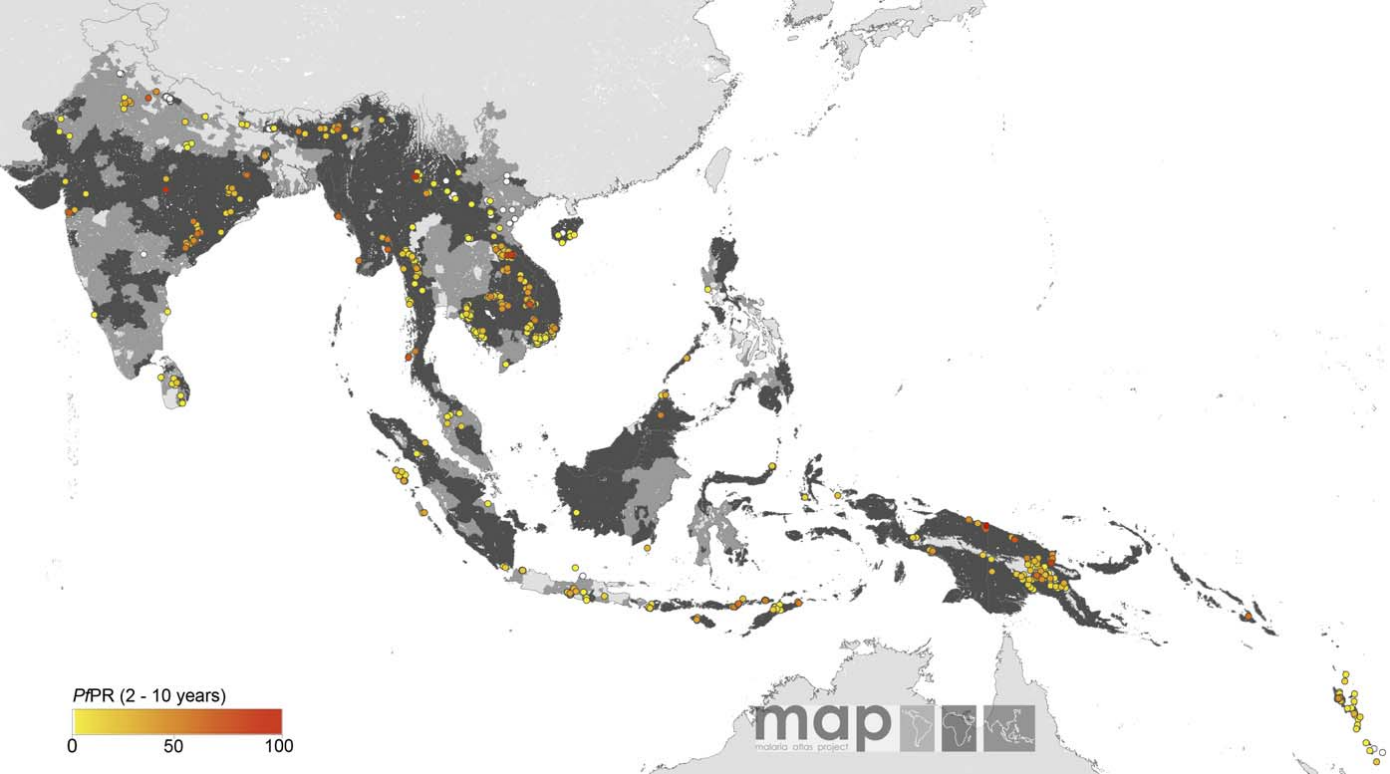
Community Surveys of P. falciparum Prevalence Conducted between 1985 and 2007 in SEARO-WPRO

The global diversity of the age-corrected *Pf*PR_2–10_ estimates within the limits of transmission is shown in Figures 2–5. A total of 253 surveys reported zero prevalence among 2,121 surveys undertaken in AFRO (Figure 2). Outside of Africa, 358 surveys reported zero prevalence among 1,565 surveys undertaken within the defined limits of transmission. Over 92% and 95% of surveys reporting *Pf*PR_2–10_ ≥ 50% and ≥ 75%, respectively, were located in AFRO and concentrated mostly between 15° latitude north and south, areas inhabited by Anopheles gambiae s.s. [54] (Figure 2). Conversely lower estimates of *Pf*PR_2–10_ were described among those surveys conducted in areas occupying the A. arabiensis–dominant regions along the Sahel, horn, and southern areas of Africa [54] (Figure 2). In AMRO (Figure 3) and EMRO-EURO (Figure 4), 87% and 65% of surveys reported *Pf*PR_2–10_ below 10%, respectively, referred to classically as hypoendemic. Over 65% of *Pf*PR_2–10_ survey estimates in the combined SEARO-WPRO region reported infection prevalence below 10% (Figure 5), including 218 surveys reporting zero prevalence.

**Table 2:**
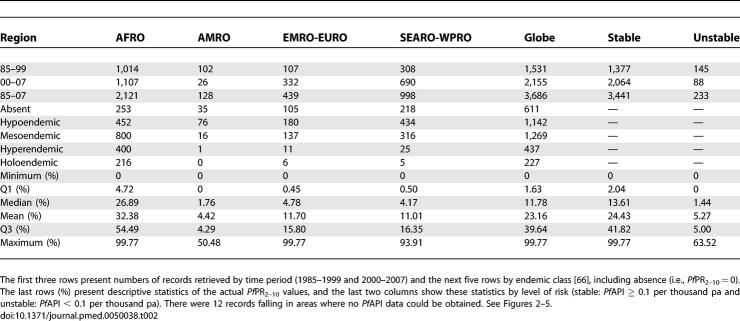
Summaries of the P. falciparum Parasite Rate Data Reported between 1985 and 2007 and Mapped within the Spatial Limits of P. falciparum Malaria

**Figure 6:**
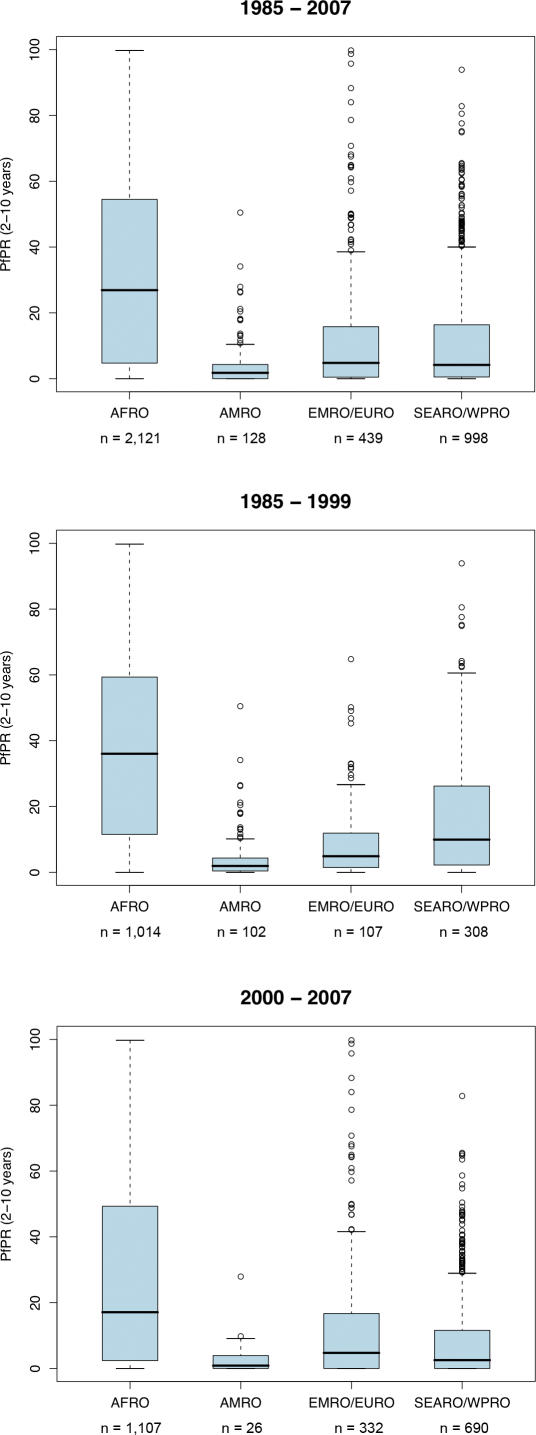
Box and Whisker Plots of *Pf*PR_2–10_ by Period and WHO Regions Thick black lines are the medians, and the light-blue boxes represent interquartile ranges; whiskers show extreme, non-outlier observations. Empty circles represent mild and/or extreme outliers. Sample sizes correspond to those shown in Table 2.

Despite notable gaps in the coverage of *Pf*PR_2–10_ data in many areas (Figures 2–5), a summary of the ranges of prevalence survey estimates is provided in Table 2 and Figure 6. These data are presented for the whole time period (Figure 6, top panel) and stratified by time (Figure 6, middle and bottom panels). We stress that these data are not spatially congruent and therefore should not be viewed as representing secular changes in *Pf*PR_2–10_ estimates by WHO region. The data used for the bottom panel of Figure 6 are potentially of greater value, however, when describing the endemicity characteristics of malaria within the spatial limits shown in Figure 1, as they represent the most contemporary summary of malaria endemicity judged by *Pf*PR_2–10_.

## Discussion

We have triangulated as much information as we could assemble from exhaustive searches to provide an improved evidence-based description of the limits of P. falciparum transmission globally. The spatial referencing of health statistics, medical intelligence, and national expert opinion represents, to our knowledge, the most complete, current framework to understand the global distribution of P. falciparum risk in 2007. The use of plausible biological constraints upon transmission, based on long-term temperature data and remotely sensed correlates of vegetation cover, improved the spatial precision of the limits and categories of risk. We estimate that there were 2.37 billion people at risk of P. falciparum worldwide in 2007, and 40.1 million km^2^ of the world might be able to support P. falciparum transmission.

Assembling geographic information on disease risk is an iterative process, building on new data and identifying gaps in our knowledge. We have presented previously the distribution of P. falciparum using historical descriptions of risk [1,16] and through the reconciliation of information in multiple travel advisories [55,56]. None have been perfect representations of contemporary malaria distributions worldwide, but such work has initiated a dialogue on the importance of providing an evidence base to malaria cartography and in the sharing of this information [15].

We have not considered the spatial distribution of P. vivax in this paper for a number of methodological reasons. First, the accuracy of health reporting systems for P. vivax clinical cases in areas of coincidental P. falciparum risk is notoriously poor [57]. Second, the climatic constraints on parasite–vector survival are less well defined and thus harder to predict using standardized regional-specific vector bionomics [58]. Third, the combined effects of a prolonged liver stage and the consequences upon natural and drug-resistant recrudescence make the interpretation of prevalence data considerably harder for P. vivax compared to P. falciparum [59]. We are acutely aware that the spatial extent and disease burden of P. vivax merits more attention than it has received, but to achieve an informed evidence-based map similar to that of P. falciparum demands a more fundamental construction of the basic biology of transmission and clinical epidemiology before this can be attempted effectively.

We have been cautious in the use of the *Pf*API data reported at national levels, recognizing the inadequacies and incompleteness of malaria surveillance [1,23–26]. The intention has been to identify administrative reporting areas that had not detected cases of P. falciparum malaria between 2002 and 2006. It was also recognized that there existed a wide range of reported *Pf*API estimates, from one case per 100,000 people pa to reports of confirmed cases in almost 50% of the population every year, which presents a problem for the classification of risk. We therefore applied threshold criteria that would distinguish areas of low clinical risk (i.e., those areas reporting few cases and likely to support unstable transmission conditions) from areas with higher reported case incidence and probably more stable in their P. falciparum transmission characteristics. Our use of a distinction between unstable and stable transmission at 0.1 per thousand pa, while conservative is not without precedent. During the era of the Global Malaria Eradication Programme, epidemiologists proposed a variety of criteria to describe malaria risk in concert with preparatory, active, consolidation, and maintenance phases of elimination and ultimate “eradication” [60–63]. Parasite prevalence was the metric of choice for defining baseline endemicity in the preparatory phase and was useful as an indicator of control progress in the attack phase [52,64], until it became impossible to measure with cost-efficient sampling at very low levels of endemicity. At this juncture, it was proposed that malaria risk be measured through incidence metrics such as the *Pf*API [65]. We identified very few *Pf*PR surveys (*n* = 233) undertaken in areas where reported *Pf*API was below 0.1 per thousand pa, 70 (30%) of which reported zero prevalence (Figures 2–5); and the median parasite prevalence was 1.4% (Table 2). It seems appropriate, practical, and feasible to consider multiple metrics during the assembly of malaria risk maps, and we have combined two common malariometric measures of risk: the *Pf*API and *Pf*PR. The mathematical relationship between these measures and other traditional epidemiological measures, such as the basic reproduction rate of infection and the entomological inoculation rate, is the subject of ongoing research [61]. Stratification of these risk areas by dominant vector species to enable a more informed assessment of the appropriate suites of intervention measures is also being pursued actively [15].

The *Pf*PR data have been assembled from peer-reviewed literature, unpublished ministry of health sources, postgraduate theses and provision of raw data from malaria scientists in all malaria endemic regions [45]. They do not derive from nationally representative, random-sample surveys. Their coverage might, therefore, be subject to bias toward areas thought to be more malarious. The inclusion of 971 geopositioned surveys reporting zero prevalence (including 523 [53.8%] from Africa), however, does not support this view.

Future investigation of the ecological and climatic covariates of *Pf*PR_2–10_ will need to move from the categorical descriptions of over-dispersed endemicity data presented here, to geostatistically robust estimates of risk that are cognisant of the many potential biases in these data across the entire limits of stable transmission shown in Figure 1. We note, however, that as infection prevalence responds to increased intervention coverage and access to effective medicines, the use of traditional biological covariates might prove less effective in predicting the distribution of P. falciparum transmission intensity. Spatial models of *Pf*PR distribution are being developed and tested as part of MAP's ongoing research to more accurately reflect the ranges of malaria transmission intensity within the margins of stable endemicity. Moreover, the *Pf*API and *Pf*PR data described in the present paper will change with time, and future data assemblies need to be maintained in a world with a rapidly changing malaria epidemiology. The supporting geostatistical models used to predict the spatial distribution of endemicity must also therefore facilitate rapid updates. The annual revision of the spatial limits of stable and unstable malaria, based upon new medical intelligence, *Pf*API summaries, and the increasingly available contemporary *Pf*PR information will iteratively redefine the cartography of malaria and be hosted on the MAP website (http://www.map.ox.ac.uk) as a public domain resource [15].

Assuming some degree of fidelity in the descriptions of unstable malaria used here, we estimate that one quarter (∼26%) of the malaria-endemic areas of the world are exposed to some degree of unstable P. falciparum transmission and home to approximately one (0.98) billion people. Even within the regions with more stable transmission, the available empirical evidence from contemporary *Pf*PR_2–10_ survey data is that outside of AFRO, the intensity of transmission is best described as hypoendemic [66] (Figure 6). This observation has important implications for how we view malaria control and broader development goals at a global scale over the next decade. The provisional categorical descriptions of global P. falciparum malaria risk are shown in Figure 1 and suggest that, at a global scale, an aggressive approach to P. falciparum elimination might be reconsidered as a more ambitious and achievable objective in many areas.

Regional initiatives aimed at elimination have begun [11–14]. In the Americas, elimination is considered in the most recent 5-y regional strategic plan [12]. In the European region, the two *Pf*MECs (Tajikistan and Kyrgyzstan) are targeted for P. falciparum elimination within the next 5 y [11,13]. Detailed plans have been developed in the Eastern Mediterranean region to consider targeted P. falciparum elimination strategies in Iran and Saudi Arabia, while strengthening maintenance phases of elimination in currently P. falciparum–free countries [14]. With the exception of EURO, detailed maps of the spatial extents of risk in these various regions are not available. Where elimination is considered a viable strategy, resource requirements, targets, and maps become a regional and sub-regional public good and are no longer solely national concerns. Saudi Arabia is providing substantial financial support for the elimination of malaria in its neighbour, Yemen [67]. If this plan is successful, the reportedly high rates of population inflow from Somalia [68] will pose a continued concern due to the potential reintroduction of the parasite. This situation further highlights the need for a reproducible and evidence-based global map of malaria risk that is maintained as a dynamic platform to estimate and predict cross-border risk.

Maintaining the detail necessary to map the spatial extent of malaria risk is paramount to the future of malaria control outside of Africa over the next 5 y. We would also argue, however, that Africa has been labelled inappropriately as a vast expanse of holoendemic transmission, intractable to control. Less than a third of all surveys retrieved from AFRO (29%) reported parasite prevalence above 50%, and, as has been described, these results followed closely the distribution of A. gambiae s.s. [54]. The conditions of hypoendemic and mesoendemic transmission were common in surveys conducted outside of this belt (which are not subject to the ravages of this most efficient vector) and are likely to benefit from approaches to prevention and control specific to the underlying ecologic and epidemiologic conditions [15,69,70]. The descriptions of transmission intensity are dynamic and the *Pf*PR_2–10_ estimates in Africa (Figure 2) do not correspond to levels of endemicity described four decades ago [17]. In the AFRO region, there has been a recent expansion of insecticide-treated net coverage and provision of effective medicines. These programmatic successes are showing tangible impacts on mortality [8,9,71] and morbidity [8,9,72], and it would seem entirely plausible that similar effects will be operating at the level of transmission. If Africa is undergoing a malaria epidemiological transition, capturing this dynamic through mapped information on infection prevalence, and planning accordingly, should be high on the control agenda.

The current focus of the Roll Back Malaria movement is, appropriately, in Africa, as this continent bears the brunt of malaria morbidity and mortality [73,74] and the descriptions presented here reinforce this view. P. falciparum transmission is a global problem, however, requiring a global strategy with regional targets and approaches tailored to what can be achieved within defined intervention periods [61]. This strategic planning demands an epidemiologically consistent map that is constantly updated. The assembly of risk data presented here represents the first attempt to combine disparate sources of malariometric data that should serve as a dynamic platform to define a global strategy and map its progress over the coming decades. The maps and national levels of population at unstable and stable risk are released in the public domain, with the publication of this paper, to further that global effort (MAP, http://www.map.ox.ac.uk).

## Supporting Information

Protocol S1 Sources and Descriptions of Medical Intelligence Used to Describe the *Pf*API (346 KB DOC)

Protocol S2 Developing Global Biological Limits for P. falciparum Transmission (1.3 MB DOC)

Table S1 National Estimates of Population at Risk of P. falciparum Malaria in 2007 (231 KB DOC)

## Abbreviations

AFRO: African Regional Office of the WHO
AMRO: American Regional Office of the WHO
API: annual parasite incidence
EMRO: Eastern Mediterranean Regional Office of the WHO
EURO: European Regional Office of the WHO
EVI: enhanced vegetation index
GIS: geographic information system
MAP: Malaria Atlas Project
pa: per annum
PAR: populations at risk
*Pf*API: *P. falciparum* annual parasite incidence
*Pf* MEC: *P. falciparum* malaria endemic country
*Pf* PR: *P. falciparum* parasite prevalence rate
*Pf* PR_2–10_: *P. falciparum* parasite prevalence rate corrected to the 2–10 y age group
SEARO: Southeast Asian Regional Office of the WHO
WHO: World Health Organization
WPRO: Western Pacific Regional Office of the WHO
