# Supplementary material for: The Limits and Intensity of Plasmodium falciparum Transmission: Implications for Malaria Control and Elimination Worldwide
Source: PLoS Med. 2008 Feb 26;5(2):e38. doi: 10.1371/journal.pmed.0050038 (PMC2253602; doi:10.1371/journal.pmed.0050038)
Supplement: Protocol S1 — (346 KB DOC) [file pmed.0050038.sd001.doc]

**Protocol S1. Methods and sources of medical intelligence used to describe *Plasmodium falciparum* annual parasite incidence (*Pf*API) globally**

We defined areas of extremely low, unstable transmission risk as spatial units reporting a *Pf*API of less than 0.1 per 1000 population per annum (pa). This is because this criterion was found to be a reliable indicator for the cessation of indoor residual spraying during the consolidation phase of the Global Malaria Eradication Programme [1-4]. During this period, the limit was reduced from 0.5‰ as it became recognized that surveillance, including passive and active case detection of incident cases, was often less accurate and reliable than nations thought: malaria often resumed after the cessation of spraying from 0.5‰, but rarely from 0.1‰. This more conservative categorization of unstable transmission around one clinical *P. falciparum* case 10,000 population (0.1‰ pa), thus, helps compensate for the vagaries of district or provincial level reporting of parasitologically confirmed cases [5-7]. Using a higher threshold between ‘stable’ and ‘unstable’ transmission would have required a greater confidence in the fidelity of the source information and the underlying surveillance.

***Pf*API data sourcing**

Table SI A1 summarizes the *Pf*API data collection process and availability for all *P. falciparum* malaria endemic countries. Complete *Pf*API data were not available for Ecuador, Bolivia, Haiti, Suriname and Venezuela. Risk maps presented at a regional malaria meeting in Cartagena, Colombia [8], were used, showing high, medium, low and no risk areas for *P. falciparum* and *P. vivax*. These maps were digitized and then combined with first-level administrative unit species-specific API risk data reported in 2002 [9] to constrain the *Pf*-limits within each country and map areas of either <0.1 or ≥0.1 cases per 1000 people pa. When these first-level administrative unit case reports were less than 10 in 2002 [9], it was assumed that contemporary risks in these areas were <0.1 ‰ pa. In Venezuela, the high risk areas reported in the Cartagena meeting [8] corresponded with descriptions of international travel advisories which state that risk in Amazonas and Bolivar is mainly restricted to the Orinoco River basin and its tributaries in the former, and along the same river in the latter but also in areas bordering the states of Apure and Guarico [10]. These descriptions were used to create a 60 km distance buffer of high risk along the Orinoco and its tributaries. The map of Venezuela in the Cartagena report shows high risk areas towards the north mainly in the state of Sucre [8]. This disagrees with published reports [9,11] of API in Sucre being attributed only to *P. vivax*, however, and probably refers to the local high risk of *P. vivax* malaria. Sucre was, therefore, classified at no risk for *P. falciparum*. For Suriname, similar descriptions of high risk restricted to the Maroni river are available [8] and, therefore, a similar 60 km buffer was created along this river and classified as high risk. The latter buffer did not capture two small high risk areas west of the river shown in the Cartagena map [8] and these were left as unstable risk due to lack of geographic information to digitize them with precision.

*Plasmodium falciparum* API data were not available for most countries within the WHO Africa region, largely because these are not regarded as priority malaria metrics for this region and reporting systems are probably the least reliable when compared to other regions of the world [5-7,12,13]. Southern Africa defined as Swaziland, South Africa, Zimbabwe, Botswana and Namibia, represents an exception with a combination of published risk data [14,15] and expert opinion from national malaria control programmes [16] used to estimate the limits of risk through matched administrative boundaries or digitized maps in these countries. For island populations risks were reviewed from published sources to confirm current *P. falciparum* transmission status including Cape Verde [17], Comoros [18], Mayotte [19], and Mauritius [19]. The islands of Reunion and Seychelles were excluded as malaria endemic as confirmed through published literature [19].

Malaria programme managers in EMRO confirmed that the small foci of malaria transmission in Ihrir, Illizi Department, Algeria and the Fayoum Governate, Egypt, are entirely due to *P. vivax*. Similarly, the limited cases reported in Syria were all due to *P. vivax*. These countries were, therefore, confirmed as *P. falciparum* free. Tajikistan and Kyrgyzstan were regarded as the only *P. falciparum* endemic countries in the WHO’s European region. Both countries have reported *P. falciparum* malaria cases since 2003 [20], with very few cases reported in 2006, however, and with elimination scheduled by 2010 [21].

Medical intelligence confirmed the *P. vivax* only status of the Korean Peninsula [22,23], Argentina [24], Paraguay [24], Iraq [25], Uzbekistan [26], Turkey [25], Azerbaijan [25] and Mauritius [27]. The United Arab Emirates have not reported any autochthonous cases of *P. falciparum* malaria for over five years [28] and satisfied the criteria for the certification of the eradication of malaria in 2007 [29]. The Chiapas region of Mexico, Canton Matua, in Costa Rica and Ahuachapa in El Salvador have all had only 1 to12 *P. falciparum* cases reported over the last five years, and these countries are regarded as largely *P. falciparum* free [24].

**Mapping *Pf*API data**

In order to map *Pf*API data consistently, digital boundaries of first and second level units were obtained from the United Nations' Second Administrative Level Boundaries (SALB) dataset (n=24) [30] and the Global Administrative Unit Layers (GAUL) developed by the Food and Agriculture Organization (FAO; n=82) [31]. In addition, *Pf*API classifications were reconciled at third-level administrative units for Nepal (Global Administrative Areas database of the University of California at Berkeley, http://biogeo.berkeley.edu/gadm) and restricted areas in Peru through the Peruvian Dirección General de Epidemiología (http://www.oge.sld.pe). In South Africa, third-level administrative data [16] were digitized against boundary data provided by FAO Geonetwork [32].

**Table 1**. Sources and spatial/temporal resolutions of reported *P. falciparum* annual pa rasite incidence (*Pf*API) data for 87 countries identified as endemic for *P. falciparum* malaria. The data are grouped by World Health Organization (WHO) region (AFRO, African Regional Office of the WHO, AMRO, American Regional Office of the WHO, EMRO, Eastern Mediterranean Regional Office of the WHO, EURO, European Regional Office of the WHO, SEARO, South East Asian Regional Office of the WHO and WPRO, Western Pacific Regional Office of the WHO) and summarised globally. Additional data sources consulted to confirm and/or refine the risk margins are also presented. ADMIN1, 2 or 3 refers to administrative division at the first, second or third levels where Admin0 is the national boundary.

| **Country** | **ADMIN level for *Pf*API^*^** | **Time period** | **Sources consulted** | **Notes** |
| --- | --- | --- | --- | --- |
|  |  |  |  |  |
| **AFRO** |  |  |  |  |
| Angola | NA | NA |  |  |
| Benin | NA | NA |  |  |
| Botswana | ADMIN1 | NA | [33-35] |  |
| Burkina Faso | NA | NA |  |  |
| Burundi | NA | NA |  |  |
| Cameroon | NA | NA |  |  |
| Cape Verde | ADMIN1 | NA | [17,33] |  |
| Central African Rep. | NA | NA |  |  |
| Chad | NA | NA |  |  |
| Comoros | ADMIN1 | NA | [18] |  |
| Congo | NA | NA |  |  |
| Côte d'Ivoire | NA | NA |  |  |
| Dem. Rep. of Congo | NA | NA |  |  |
| Equatorial Guinea | NA | NA |  |  |
| Eritrea | NA | NA |  |  |
| Ethiopia | NA | NA |  |  |
| Gabon | NA | NA |  |  |
| Gambia | NA | NA |  |  |
| Ghana | NA | NA |  |  |
| Guinea | NA | NA |  |  |
| Guinea-Bissau | NA | NA |  |  |
| Kenya | NA | NA |  |  |
| Liberia | NA | NA |  |  |
| Madagascar | NA | NA | [36] |  |
| Malawi | NA | NA |  |  |
| Mali | NA | NA |  |  |
| Mauritania | NA | NA | [33] |  |
| Mayotte | ADMIN1 | NA | [37] |  |
| Mozambique | NA | NA |  |  |
| Namibia | ADMIN2 | NA | [33,35,38] |  |
| Niger | NA | NA |  |  |
| Nigeria | NA | NA |  |  |
| Rwanda | NA | NA |  |  |
| Sao Tome and Principe | NA | NA |  |  |
| Senegal | NA | NA |  |  |
| Sierra Leone | NA | NA |  |  |
| South Africa | ADMIN2 | NA | [15,16,35] |  |
| Swaziland | ADMIN2 | NA | [35] |  |
| Togo | NA | NA |  |  |
| Uganda | NA | NA |  |  |
| United Rep.of Tanzania | NA | NA |  |  |
| Zambia | NA | NA |  |  |
| Zimbabwe | ADMIN2 | NA | [33,35,39,40] |  |
|  |  |  |  |  |
| **AMRO** |  |  |  |  |
| Belize | ADMIN2 (6) | 2004 |  |  |
| Bolivia | ADMIN2 (112) | 2002 | [8,9,33,41] |  |
| Brazil | ADMIN2 (5310) | 2004-2006 | [8] |  |
| Colombia | ADMIN2 (297) | 2005 | [8,33] |  |
| Dominican Rep. | ADMIN1 (32) | 2004 |  |  |
| Ecuador | ADMIN2 (220) | 2002 | [8,9,33] |  |
| French Guiana | ADMIN2 (21) | 2006 | [33] |  |
| Guatemala | ADMIN1 (22) | 2004 |  |  |
| Guyana | ADMIN1 (10) | 2004 |  |  |
| Haiti | ADMIN2 (41) | 2002 | [33] |  |
| Honduras | ADMIN1 (19) | 2004-2006 |  |  |
| Nicaragua | ADMIN1 (18) | 2004 |  |  |
| Panama | ADMIN2 (68) | 2006 |  |  |
| Peru | ADMIN2 (191) and ADMIN3 (14) | 2004 | [8,33] |  |
| Suriname | ADMIN2 (62) | 2002 | [9,33] | A 60km buffer around the Maroni river was assumed to correspond to high risk |
| Venezuela | ADMIN2 (318) | 2002 | [9,33,41] | Assumptions of risk confined mainly near the Orinoco and its main tributaries (60km buffer) were deemed sensible to avoid overestimating risk in Bolivar and Amazonas |
|  |  |  |  |  |
| **EMRO** |  |  |  |  |
| Afghanistan | ADMIN1 (32) | 2005 | [42] |  |
| Djibouti | ADMIN1 | NA | [43] |  |
| Iran | ADMIN2 (251) | 2004-2006 | [44,45] |  |
| Pakistan | ADMIN2 (119) | 2004-2006 | [46] | No information available for six ‘tribal areas’ in the Fata region of Pakistan and 15 ADMIN1 units in the disputed territory of Jammu Kashmir. |
| Saudi Arabia | ADMIN1 (13) | 2005-2006 | [47] |  |
| Somalia | ADMIN2 | NA |  |  |
| Sudan | ADMIN1 | NA |  |  |
| Yemen | ADMIN1 (19) | 2002, 2005-2006 |  |  |
| **EURO** |  |  |  |  |
| Kyrgyzstan | ADMIN2 (40) | 2002-2005 | [20,48] |  |
| Tajikistan | ADMIN2 (56) | 2005-2006 | [20,48] |  |
|  |  |  |  |  |
| **SEARO** |  |  |  |  |
| Bangladesh | ADMIN2 (64) | 2003-2004 | [49] |  |
| Bhutan | ADMIN1 (20) | 2002-2004 | [33,49] |  |
| India | ADMIN2 (538) | 2002-2004 |  | Twenty-two ADMIN1 units could not be reconciled or matched to any reported *Pf*API data in India and were left as missing data. |
| Indonesia | ADMIN1/2 (281) | 2005 | [49] | The following rules were used to interpolate ADMIN1 data to some ADMIN2 polygons: i) in 5 cases, no ADMIN2 data were available for the whole ADMIN1 and this was defaulted to 2005 ADMIN1 data; ii) there were 50 ADMIN2s for which no data were available but other ADMIN2 units within the same ADMIN1 had data; these missing ones were assigned the overall ADMIN1 value. |
| Myanmar | ADMIN1 (14) and ADMIN2 (11) | 2003-2004 | [33,49] |  |
| Nepal | ADMIN3 (175) | 2002-2003 | [33,49] |  |
| Sri Lanka | ADMIN2 (25) | 2004 | [33,50] |  |
| Thailand | ADMIN1 (76) | 2003-2004 | [33,51] |  |
| Timor Leste | ADMIN1 (13) | 2004-2005 |  |  |
|  |  |  |  |  |
| **WPRO** |  |  |  |  |
| Cambodia | ADMIN1 (24) | 2003-2005 |  |  |
| China | ADMIN1 (32) ADMIN2 (16)^i^ | 2003-2005 | [33,52-54] |  |
| Lao Dem. Peoples Rep. | ADMIN1 (18) | 2003-2005 | [33,55] |  |
| Malaysia | ADMIN1 (15) | 2003-2005 | [56] |  |
| Papua New Guinea | ADMIN1 (20) | 2003-2005 | [57] |  |
| Philippines | ADMIN2 (79) | 2003-2005 | [33] |  |
| Solomons | ADMIN1 (10) | 2003-2005 |  |  |
| Vanuatu | ADMIN1 (6) | 2003-2005 |  |  |
| Vietnam | ADMIN1 (61)^i^ | 2003-2005 | [33,55] |  |

^*^Missing data: Apart from data missing in Pakistan and India (specified in the table), data were not available for the following territories: The Santanilla islands of Honduras, D.I. Yogyakarta of Indonesia, the district of Kilinochchi in Sri Lanka and the province of Bac Nihn in Vietnam.

**References**
