# Supplementary material for: The Limits and Intensity of Plasmodium falciparum Transmission: Implications for Malaria Control and Elimination Worldwide
Source: PLoS Med. 2008 Feb 26;5(2):e38. doi: 10.1371/journal.pmed.0050038 (PMC2253602; doi:10.1371/journal.pmed.0050038)
Supplement: Protocol S2 — (1.3 MB DOC) [file pmed.0050038.sd002.doc]

**Protocol S2: Developing global biological limits for *Plasmodium falciparum* transmission**

**The temperature mask**

Temperature affects many aspects of mosquito physiology [1]. One aspect critical for malaria transmission is the temperature dependence of sporogony: the time it takes for sporozoites to develop and become infectious in *Anopheles*. A method for estimating the duration of sporogony has been proposed, based on the number of degree-days required by the parasite to complete development [2,3], for malaria the sum of the number of degrees in a day by which the mean temperature exceeds the minimum required for the development of sporozoites. Nikolaev [4] showed that the degree-days required for the maturation of sporozoites in an *An. maculipennis* population from Russia were 105 for *P. vivax*, 111 for *P. falciparum* and 144 for *P. malariae* and that parasite development ceased below 16^o^C for *P. falciparum* and *P. malariae* and below 14.5^o^C for *P. vivax*. The duration of sporogony (DS) in days can thus be calculated as:

[1]

where *DD* are the parasite species-specific degree-days, *T_MEAN_* is the mean ambient temperature and *T_MIN_* is the minimum temperature required for parasite development.

Figure 1 plots the results of the above equation for *P. falciparum*, *P. vivax* and *P. malariae* development in *An. maculipennis*. It shows that *P. vivax* is able to develop at the lowest temperatures, followed by *P. falciparum* and *P.* malariae, thus helping explain the species specific latitudinal limits of the parasites globally [5,6]. The figure also shows that the length of sporogony of *P. falciparum* increases rapidly when temperatures drop below 22^o^C. The curve never reaches a true asymptote on the *y-*axis; but the duration of sporogony becomes so extended that few anophelines will survive long enough to inoculate humans and at 16^o^C parasite development ceases entirely. Conversely, as temperature increases the duration of sporogony decreases, so that at 30^o^C it can take less than ten days. Obviously, the duration of sporogony then becomes limited by parasite and vector survival, which plummet as temperatures rise above 32^o^C [7]. The duration of sporogony is dependent fundamentally on enzyme kinetics [8] and thus widely assumed to be relatively independent of vector species. It is the interplay between the duration of sporogony and the species-specific longevity of the *Anopheles* vector that forms the basis of the temperature mask.


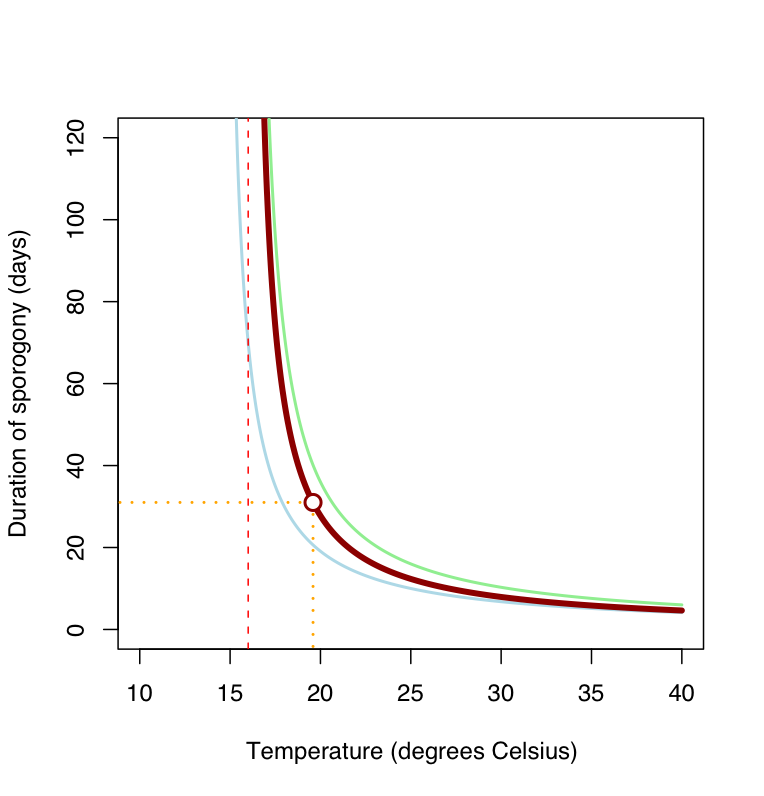


**Protocol S2, Figure 1**. The relationship between the duration of sporogony and temperature for *P. falciparum* (dark red), *P. vivax* (blue) and *P. malariae* (green). The open circle indicates the temperature below which the length of sporogony for *P. falciparum* is not outlived by most vectors, which corresponds to 31 days at *circa* 19.58^o^C (dotted orange lines). The dashed red line indicates the absolute temperature below which *P. falciparum* development ceases at 16^o^C.

Kiszewski *et al.* [9] provide a comprehensive review of the longevity of *Anopheles* from natural and experimental studies of the bionomics of 33 malaria vectors worldwide that were viewed to be dominant contributors to local transmission. The resulting summary of daily survival rates for adult anophelines was found to be so variable that the authors used a median value for all vectors for their malaria stability index. The mean daily survival rate was 0.846 for all *Anopheles*, ranging from 0.682 for *An. albimanus* to 0.950 for *An. sergentii*.

We have further limited the criteria for dominant vector species by insisting that they were indicated as dominant in local malaria transmission by all four of the following authors [9-13]. The daily survival rate for the 19 main/dominant vectors by region was used to determine the fraction of the population surviving over successive days (Figure 2). Although temperature will affect other parameters of the basic reproduction rate of infection [14], including biting and resting vector habits, it seems reasonable to consider the proportion of the population surviving 31 days as the critical point of interruption of *P. falciparum* transmission. To a close approximation, most vector populations would have been reduced to 99% of their original population size within 31 days. The regional variation encountered in this approximation was then explored further.

In AMRO, the mean daily survival probability for the five regionally dominant vectors is 0.782. This means that 99.95% of an initial population would not survive 31 days (Figure 2, top left). By far the longest-lived vector in the region is *An. pseudopunctipennis* for which 98.09% of the population will have perished in 31 days; this makes this vector resistant to higher altitudes across its distribution in the Andean slopes [15]. Similarly, the mean daily survival probability for the three regionally dominant vectors in AFRO is 0.780. This means that 99.95% of an initial population would not survive 31 days (Figure 2, top right). In SEARO/WPRO the mean daily survival probability for the eight regionally dominant vectors is 0.836. This predicts that 99.62% of an initial population would not survive 31 days (Figure 2, bottom right). The longest lived vector in the region is *An. dirus*, for which 93.41% of the population will have perished in 31 days. This species is mainly a forest dweller [16,17] and therefore is restricted geographically and ecologically to a niche that does not suffer extreme temperature limits. Excluding areas where average temperatures were such that sporogony would not complete in 31 days was thus considered a biologically plausible and conservative limit of *P. falciparum* malaria transmission in these regions.

In EMRO the mean daily survival probability for the five regionally dominant vectors is 0.846. This indicates that 99.45% of an initial population would not survive 31 days (Figure 2, bottom left). This mean disguises two relatively long-lived vectors in the region: *An. sergentii*, which is an oasis breeder, and *An. superpictus*, which is another mountain foothill breeder [11,12,18]. These species have longevities that predict that only 79.61% and 82.69% of their populations would have died after a 31-day period. The 31 days time limit was applied for this region, except where these species are dominant, where it was doubled to 62 days. This duration would result in a population mortality of 95.84% and 97.00% for *An. sergentii* and *An. superpictus*, respectively. The distributions of these vectors described by White [13] were digitized and the 62-day rule applied within these species ranges.


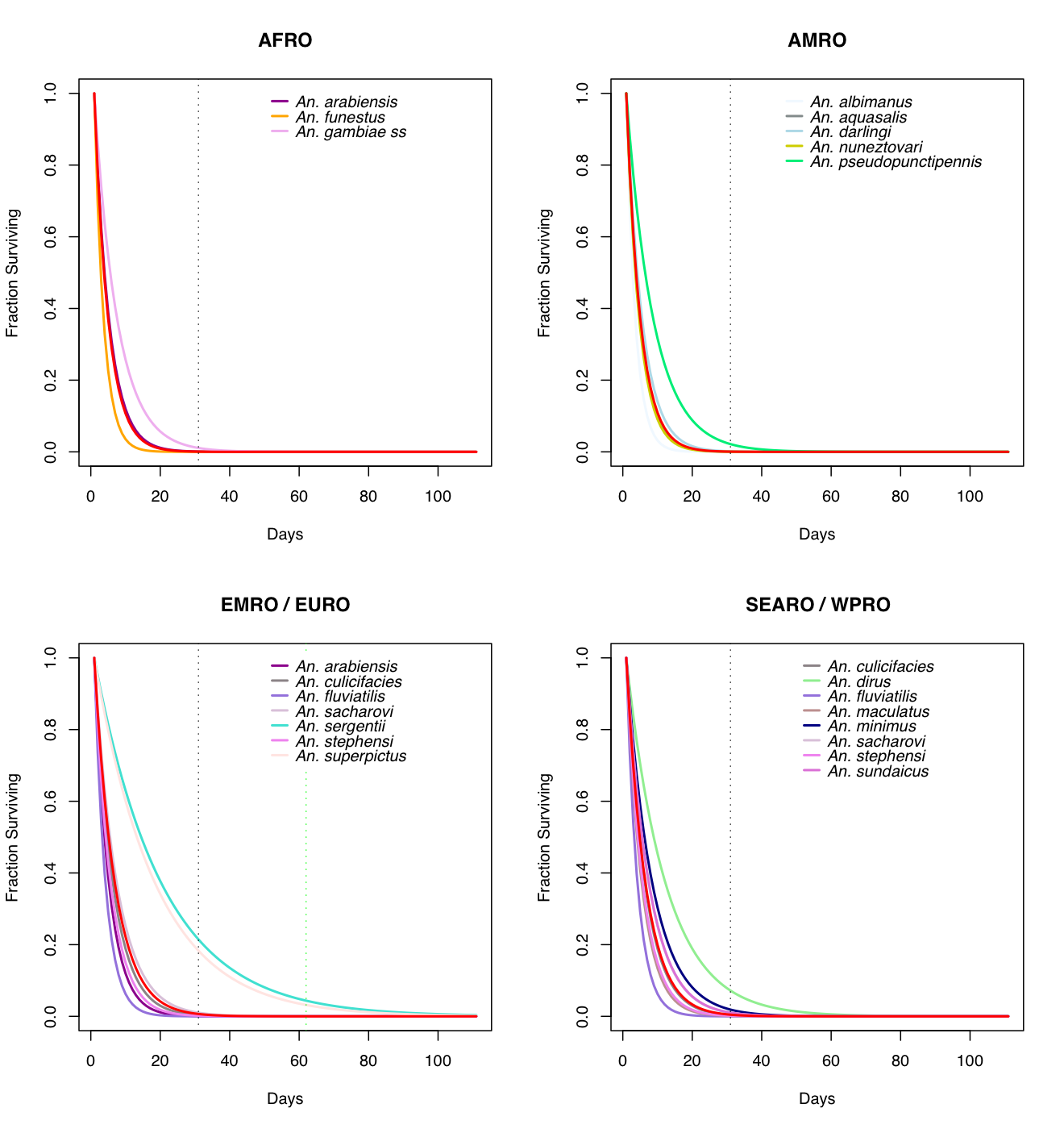


**Protocol S2, Figure 2**. The fraction of the original population of *Anopheles* surviving by day for each dominant vector species of the World Health Organization (WHO) geographical regions. (AFRO, African Regional Office of the WHO, AMRO, American Regional Office of the WHO, EMRO, Eastern Mediterranean Regional Office of the WHO, EURO, European Regional Office of the WHO, SEARO, South East Asian Regional Office of the WHO and WPRO, Western Pacific Regional Office of the WHO). In all panels, the red line is the average survival fraction for the region and each species is identified by a unique colour shown in the top right of the panel. The black dotted lines mark the 31 days duration of sporogony limit. The green dotted line in the EMRO/EURO panel marks the 62 days criterion applied for *An. sergentii* and *An. superpictus*.

In summary, with the exception of *An. sergentii* and *An. superpictus*, it is rare for adult dominant vectors of malaria to survive longer than a month, with more than 99% of the average population dying after 31 days. The longer-lived vectors are generally those adapted to survive at higher altitudes or harsher conditions, such as is the case of *An. superpictus* and *An. sergentii*. Despite the fact that a relatively small proportion of the populations of these vectors are normally able to survive longer than one month, the numbers of individuals surviving might still pose a significant risk for malaria transmission by being able to support parasite development at lower temperatures. After two months, however, most individuals of both species (>95%) would also have succumbed.

Using average monthly temperature records estimated from a global climate surface [19], the duration of *P. falciparum* sporogony was estimated for each month. Those pixels where the duration of sporogony was 31 days or less were identified in each month. This provided 12 images with a binary outcome of whether *P. falciparum* sporogony could be completed in more or less than 31 days. The images were combined to identify the number of temperature suitable months available in a synoptic year (Figure 3). All pixels where the 31 days limit was not achieved for any single month (i.e. grey pixels in Figure 3), or two consecutive months in the geographic range of *An. sergentii* and *An. superpictus*, were used as a conservative mask to exclude areas where transmission is highly unlikely to occur.


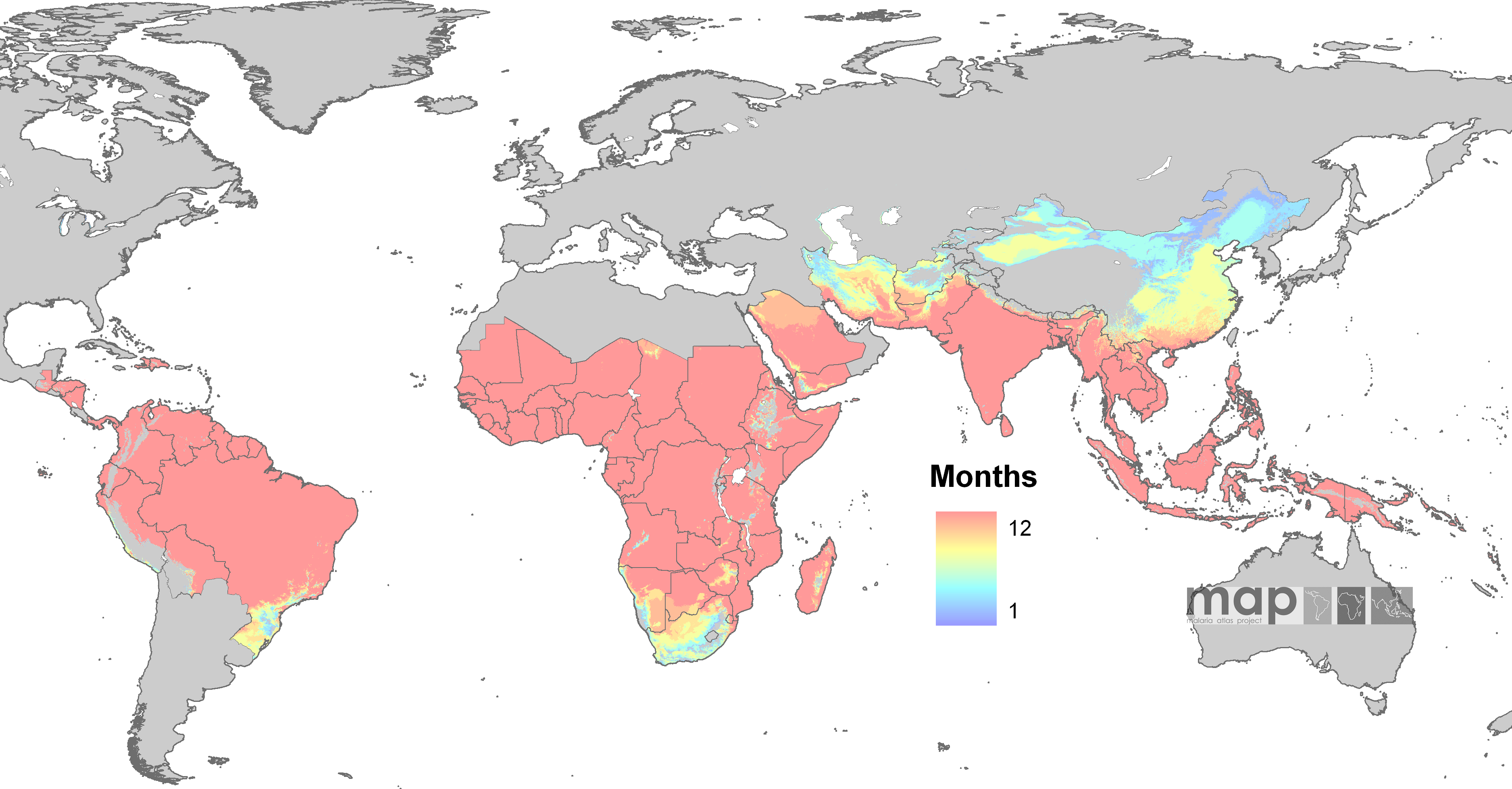


**Protocol S2, Figure 3**. An overlay of 12 monthly images of where the duration of sporogony exceeds 31 days, restricted to *P. falciparum* Malaria Endemic Countries (*Pf*MECs). Pixels where the temperature did not reach 19.58^o^C in any single month of a synoptic year (here shown as grey areas within the *Pf*MECs) were used to mask in all areas (except within the range of *An. sergentii* and *An. superpictus* were two consecutive months were required).

**The aridity mask**

Upper temperature limits were not defined on the basis of physiological tolerances of vectors measured in laboratories [20-23] as these were so high as to be rarely achieved in nature and often subject to behavioural avoidance [20]. We preferred a partial surrogate for extreme aridity: a hybrid measure encompassing both high temperature and low water availability.

The ability of adult vectors to survive long enough to contribute to parasite transmission and their eggs and larvae to survive in sufficient numbers to sustain transmission is dependent on the level of humidity and the species-specific ability to withstand arid conditions [24-26]. Hyper-aridity is the main criterion used to define a desert biome [27] and, therefore, identifying desert extents was assumed to be an accurate proxy for the extreme mask to limit the risk of malaria transmission.

These potentially limiting conditions prevail in deserts and their fringes found in malaria endemic countries, notably the Sahara (and the Sahel), the Namib, the Arabian and the Thar deserts, as well as the northern arid areas of East Africa and Peru (Figure SI B4). Since in these areas optimum growth of most plants is hindered, a proxy for vegetation cover can be used to classify arid areas [28]. Such a proxy can be derived from satellite sensors by combining the information of different channels of the electromagnetic spectrum to derive vegetation indices [29]. One of the most commonly used is the normalized difference vegetation index (NDVI) [30], available as a multitemporal series from the Advanced Very High Resolution Radiometer sensor [31] and, more recently, from the MODerate-resolution Imaging Spectroradiometer (MODIS) sensor on board the Terra and Aqua satellites [32-34].

In addition to NDVI, MODIS products include the enhanced vegetation index (EVI) [34]. EVI is calculated similarly to NDVI, which is derived from two channels of the electromagnetic spectrum (red and near-infrared). EVI incorporates a third channel (blue) and corrects for some of the distortion caused by atmospheric particles and ground cover beneath the vegetation. This makes EVI a more robust index by offering improved sensitivity, particularly in areas with high biomass content where it saturates less than NDVI, but also reduced contamination throughout by particles in the air [32-34].

Temporal Fourier processed, monthly, bi-directional reflectance distribution function corrected EVI images [32,35,36] were reclassified using ArcView GIS 3.2 (ESRI 1999) to give a binary output of areas where EVI ≤0.1 and EVI >1. These reclassified images were then overlaid in pairs to produce 12 new images. The 12 pairs were then combined to identify pixels where conditions were suitable for transmission (i.e. where EVI pixel values in a synoptic year were higher than 0.1 for at least 2 consecutive months).

Despite strict quality control, these data are affected by atmospheric contamination in the form of clouds and aerosols, although these effects are less frequent in arid low rainfall areas with infrequent cloud cover. To avoid the introduction of these errors in the final mask, a sub-mask was used of only those territories within *P. falciparum* endemic countries that were defined as being at some level of risk according to *Pf*API data and that overlapped with the arid areas defined by the EVI threshold. This included whole or partial territories of 20 *Pf*MECs as follows: Afghanistan, Angola, Chad, Djibouti, Eritrea, Ethiopia, India (northwest), Iran, Kenya, Kyrgyzstan, Mali, Mauritania, Niger, Pakistan, Peru (northwest), Saudi Arabia (southwest), Somalia, Sudan, Tajikistan and Yemen.

The aridity sub-mask was applied in a step-wise fashion by which risk was down-regulated one class (i.e. stable to unstable and unstable to no risk). Therefore, the only areas where risk was excluded were those where *Pf*API had already defined limited risk of malaria. The sub-mask was then applied on top of the *Pf*API and temperature masks (Figure 1, bottom panel, main paper).


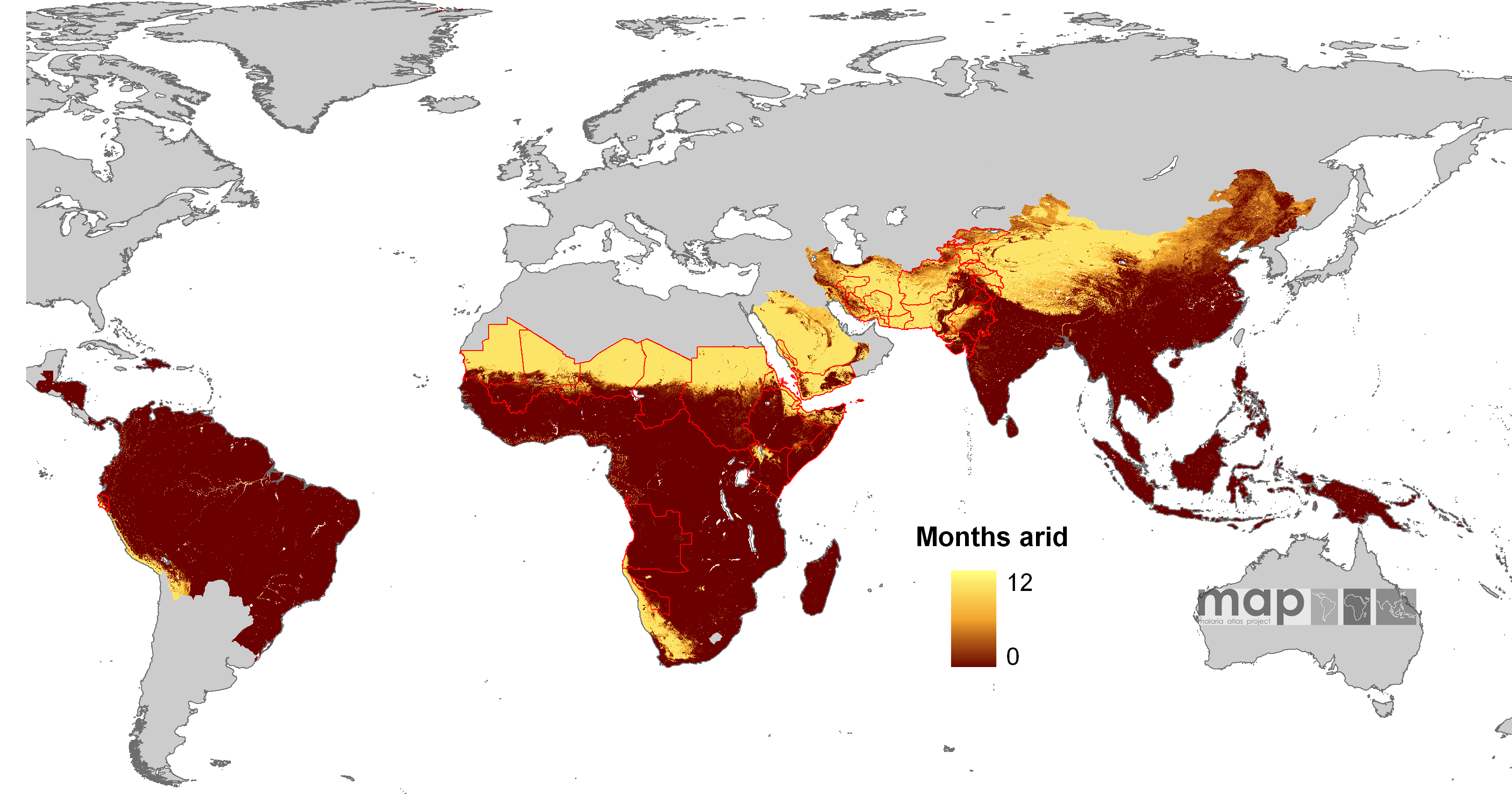


**Protocol S2, Figure 4**. Overlay of reclassified monthly EVI images (≤0.1 and >0.1). The scale shows the number of ‘arid’ months occurring in each pixel in a synoptic year. Despite quality control, cloud contamination was still evident in some humid areas (e.g. Gulf of Guinea) as fine speckle. The red outline indicates *P. falciparum* risk areas where the aridity mask was applied to avoid introducing cloud-contaminated pixels in the final image.

**References**

1. Clements AN (1999) The Biology of Mosquitoes. Wallingford, UK: CABI Publishing. 740 p.

2. Detinova TS (1962) Age grouping methods in *Diptera* of medical importance with special reference to some vectors of malaria. Geneva: World Health Organization.

3. Moshkovsky SD (1946) [The dependence upon temperature of the speed of development of malaria plasmodia in the mosquito]. Med Parazitol (Mosk) 15: 19.

4. Nikolaev BP (1935) The influence of temperature on the development of malaria plasmodia in the mosquito. Tr Pasteur Inst Epidem Bakt (Leningr) 2: 108-109.

5. Lysenko AY, Semashko IN (1968) Geography of Malaria: a medico-geographic profile of an ancient disease. In: Lebedew AW, editor. Medicinskaja Geografija. Moscow: Academy of Sciences. pp. 25-146.

6. Lysenko AJ, Beljaev AE (1969) An analysis of the geographical distribution of *Plasmodium ovale*. Bull World Health Organ 40: 383-394.

7. Muirhead-Thompson RC (1951) Mosquito behaviour in relation to malaria transmission and control in the tropics. London: Edward Arnold & Co.

8. Sharpe PJ, DeMichele DW (1977) Reaction kinetics of poikilotherm development. J Theor Biol 64: 649-670.

9. Kiszewski A, Mellinger A, Spielman A, Malaney P, Sachs SE, et al. (2004) A global index representing the stability of malaria transmission. Am J Trop Med Hyg 70: 486-498.

10. Mouchet J, Carnevale P, Coosemans M, Julvez J, Manguin S, et al. (2004) Biodiversité du paludisme dans le monde. Paris: John Libbey Eurotext. 428 p.

11. Service MW (1993) Mosquitoes (Culicidae). In: Lane RP, Crosskey RW, editors. Medical Insects and Arachnids. London: Chapman & Hall. pp. 120-240.

12. Service MW (1993) The *Anopheles* vector. Bruce-Chwatt's Essential Malariology. London: Edward Arnold. pp. 96-123.

13. White GB (1989) Malaria. In: Sloof R, editor. Geographical distribution of arthropod-borne diseases and their principal vectors WHO/VBC/89967. Geneva: World Health Organization, Division of Vector Biology and Control. pp. 7-22.

14. Rogers DJ, Randolph SE (2006) Climate change and vector-borne diseases. Adv Parasitol 62: 345-381.

15. Rubio-Palis Y, Zimmerman RH (1997) Ecoregional classification of malaria vectors in the neotropics. J Med Entomol 34: 499-510.

16. Guerra CA, Snow RW, Hay SI (2006) A global assessment of closed forests, deforestation and malaria risk. Ann Trop Med Parasitol 100: 189-204.

17. Obsomer V, Defourny P, Coosemans M (2007) The *Anopheles dirus* complex: spatial distribution and environmental drivers. Malar J 6: 26.

18. Zahar AR (1984) Vector control operations in the African context. Bull World Health Organ 62 Suppl: 89-100.

19. Hijmans RJ, Cameron SE, Parra JL, Jones PG, Jarvis A (2005) Very high resolution interpolated climate surfaces for global land areas. Int J Climatol 25: 1965-1978.

20. Kirby MJ, Lindsay SW (2004) Responses of adult mosquitoes of two sibling species, *Anopheles arabiensis* and *A. gambiae s.s*. (Diptera: Culicidae), to high temperatures. Bull Entomol Res 94: 441-448.

21. Bayoh MN, Lindsay SW (2003) Effect of temperature on the development of the aquatic stages of *Anopheles gambiae* *sensu stricto* (Diptera: Culicidae). Bull Entomol Res 93: 375-381.

22. Bayoh MN, Lindsay SW (2004) Temperature-related duration of aquatic stages of the Afrotropical malaria vector mosquito *Anopheles gambiae* in the laboratory. Med Vet Entomol 18: 174-179.

23. Jepson WF, Moutia A, Courtois C (1947) The malaria problem in Mauritius: the bionomics of Mauritian anophelines. Bull Entomol Res 38: 177-208.

24. Shililu JI, Grueber WB, Mbogo CM, Githure JI, Riddiford LM, et al. (2004) Development and survival of *Anopheles gambiae* eggs in drying soil: influence of the rate of drying, egg age, and soil type. J Am Mosq Control Assoc 20: 243-247.

25. Omer SM, Cloudsley-Thompson JL (1970) Survival of female *Anopheles gambiae* Giles through a 9-month dry season in Sudan. Bull World Health Organ 42: 319-330.

26. Omer SM, Cloudsley-Thomson JL (1968) Dry season biology of *Anopheles gambiae* Giles in the Sudan. Nature 217: 879-880.

27. UNEP (2006) Global Deserts Outlook. Nairobi: Division of Early Warning and Assessment (DEWA), United Nations Environment Programme. 184 p.

28. Suzuki R, Xu JQ, Motoya K (2006) Global analyses of satellite-derived vegetation index related to climatological wetness and warmth. Int J Climatol 26: 425-438.

29. Myneni RB, Maggion S, Iaquinto J, Privette JL, Gobron N, et al. (1995) Optical remote-sensing of vegetation - modeling, caveats, and algorithms. Remote Sens Environ 51: 169-188.

30. Tucker CJ (1979) Red and photographic infrared linear contributions for monitoring vegetation. Remote Sens Environ 8: 127-150.

31. Hay SI (2000) An overview of remote sensing and geodesy for epidemiology and public health application. Adv Parasitol 47: 1-35.

32. Hay SI, Tatem AJ, Graham AJ, Goetz SJ, Rogers DJ (2006) Global environmental data for mapping infectious disease distribution. Adv Parasitol 62: 37-77.

33. Guerra CA, Hay SI (2005) Remote Sensing: Generalities and Data Products for Malaria Risk Mapping in the Americas. In: Confalonieri UEC, Marinho DP, editors. Remote Sensing and the Control of Infectious Diseases: Proceedings from an Interamerican Workshop. Rio de Janeiro: ENSP/FIOCRUZ. pp. 71-89.

34. Tatem AJ, Goetz SJ, Hay SI (2004) Terra and Aqua: new data for epidemiology and public health. Int J Appl Earth Obs 6: 33-46.

35. Scharlemann J, Benz D, Hay SI, Purse B, Tatem AJ, et al. (2008) Global data for ecology and epidemiology: a novel algorithm for temporal Fourier processing MODIS data. PLoS One: in press.

36. Rogers DJ (2000) Satellites, space, time and the African Trypanosomiases. Adv Parasitol 47: 128-171.
