# Supplementary material for: The Limits and Intensity of Plasmodium falciparum Transmission: Implications for Malaria Control and Elimination Worldwide
Source: PLoS Med. 2008 Feb 26;5(2):e38. doi: 10.1371/journal.pmed.0050038 (PMC2253602; doi:10.1371/journal.pmed.0050038)
Supplement: Table S1 — (231 KB DOC) [file pmed.0050038.st001.doc]

**Table S1**. National level estimates of the area occupied by and number of people at risk of *Plasmodium falciparum* malaria transmission in 2007. The data are presented for World Health Organization (WHO) region: AFRO, African Regional Office of the WHO, AMRO, American Regional Office of the WHO, EMRO, Eastern Mediterranean Regional Office of the WHO, EURO, European Regional Office of the WHO, SEARO, South East Asian Regional Office of the WHO and WPRO, Western Pacific Regional Office of the WHO. Areas are in millions km^2^ and populations at risk in millions. The populations are further stratified as those at stable (*P. falciparum* annual parasite incidence (*Pf*API) of more than 0.1 per 10S00 population per annum (pa)) and unstable (*Pf*API < 0.1‰ pa).

| **Region/Country** | **Area at risk (km^2^)** | | | **Population at risk (millions)** | | |
| --- | --- | --- | --- | --- | --- | --- |
|  | **Stable** | **Unstable** | **All** | **Stable** | **Unstable** | **All** |
|  |  |  |  |  |  |  |
| **AFRO** |  |  |  |  |  |  |
| Angola | 1.26 | 0.02 | 1.28 | 15.41 | 0.28 | 15.70 |
| Benin | 0.12 | 0.00 | 0.12 | 7.66 | 0.00 | 7.66 |
| Botswana | 0.43 | 0.00 | 0.43 | 0.89 | 0.00 | 0.89 |
| Burkina Faso | 0.28 | 0.00 | 0.28 | 14.23 | 0.00 | 14.23 |
| Burundi | 0.02 | 0.00 | 0.02 | 5.66 | 0.00 | 5.66 |
| Cameroon | 0.47 | 0.00 | 0.47 | 16.95 | 0.00 | 16.95 |
| Cape Verde | 0.00 | 0.00 | 0.00 | 0.00 | 0.25 | 0.25 |
| Central African Republic | 0.63 | 0.00 | 0.63 | 4.15 | 0.00 | 4.15 |
| Chad | 0.69 | 0.63 | 1.32 | 9.76 | 0.16 | 9.92 |
| Comoros | 0.00 | 0.00 | 0.00 | 0.59 | 0.00 | 0.59 |
| Congo | 0.34 | 0.00 | 0.34 | 3.33 | 0.00 | 3.33 |
| Côte d'Ivoire | 0.33 | 0.00 | 0.33 | 17.80 | 0.00 | 17.80 |
| Dem. Republic of the Congo | 2.32 | 0.00 | 2.32 | 57.97 | 0.00 | 57.97 |
| Equatorial Guinea | 0.03 | 0.00 | 0.03 | 0.52 | 0.00 | 0.52 |
| Eritrea | 0.07 | 0.06 | 0.13 | 3.33 | 0.95 | 4.28 |
| Ethiopia | 0.93 | 0.07 | 0.99 | 46.08 | 1.50 | 47.59 |
| Gabon | 0.27 | 0.00 | 0.27 | 1.34 | 0.00 | 1.34 |
| Gambia | 0.01 | 0.00 | 0.01 | 1.51 | 0.00 | 1.51 |
| Ghana | 0.24 | 0.00 | 0.24 | 22.21 | 0.00 | 22.21 |
| Guinea | 0.25 | 0.00 | 0.25 | 9.23 | 0.00 | 9.23 |
| Guinea-Bissau | 0.03 | 0.00 | 0.03 | 1.43 | 0.00 | 1.43 |
| Kenya | 0.00 | 0.03 | 0.03 | 25.61 | 0.18 | 25.79 |
| Liberia | 0.10 | 0.00 | 0.10 | 3.43 | 0.00 | 3.43 |
| Madagascar | 0.61 | 0.09 | 0.70 | 17.28 | 0.00 | 17.28 |
| Malawi | 0.12 | 0.00 | 0.12 | 13.45 | 0.00 | 13.45 |
| Mali | 0.65 | 0.67 | 1.32 | 13.46 | 0.47 | 13.93 |
| Mauritania | 0.28 | 0.53 | 0.81 | 0.93 | 0.40 | 1.33 |
| Mayotte | 0.00 | 0.00 | 0.00 | 0.00 | 0.27 | 0.27 |
| Mozambique | 0.82 | 0.00 | 0.82 | 21.06 | 0.00 | 21.06 |
| Namibia | 0.35 | 0.19 | 0.54 | 1.25 | 0.38 | 1.64 |
| Niger | 0.45 | 0.79 | 1.25 | 13.19 | 0.62 | 13.81 |
| Nigeria | 0.93 | 0.00 | 0.93 | 134.60 | 0.00 | 134.60 |
| Rwanda | 0.02 | 0.00 | 0.02 | 5.03 | 0.00 | 5.03 |
| Sao Tome and Principe | 0.00 | 0.00 | 0.00 | 0.13 | 0.00 | 0.13 |
| Senegal | 0.20 | 0.00 | 0.20 | 10.82 | 0.00 | 10.82 |
| Sierra Leone | 0.07 | 0.00 | 0.07 | 5.50 | 0.00 | 5.50 |
| South Africa | 0.07 | 0.05 | 0.12 | 3.44 | 2.95 | 6.39 |
| Swaziland | 0.01 | 0.00 | 0.01 | 0.23 | 0.00 | 0.23 |
| Togo | 0.06 | 0.00 | 0.06 | 5.45 | 0.00 | 5.45 |
| Uganda | 0.24 | 0.00 | 0.24 | 27.03 | 0.00 | 27.03 |
| United Republic of Tanzania | 0.92 | 0.00 | 0.92 | 39.84 | 0.00 | 39.84 |
| Zambia | 0.78 | 0.00 | 0.78 | 11.84 | 0.00 | 11.84 |
| Zimbabwe | 0.31 | 0.00 | 0.31 | 7.44 | 0.00 | 7.44 |
| **Total** | **15.70** | **3.11** | **18.81** | **601.06** | **8.43** | **609.49** |
|  |  |  |  |  |  |  |
| **AMRO** |  |  |  |  |  |  |
| Belize | 0.00 | 0.01 | 0.01 | 0.00 | 0.17 | 0.17 |
| Bolivia | 0.12 | 0.56 | 0.68 | 0.22 | 2.61 | 2.83 |
| Brazil | 4.15 | 0.41 | 4.56 | 12.79 | 16.69 | 29.47 |
| Colombia | 0.52 | 0.31 | 0.83 | 5.26 | 7.74 | 13.00 |
| Dominican Republic | 0.02 | 0.01 | 0.03 | 1.41 | 2.83 | 4.24 |
| Ecuador | 0.11 | 0.07 | 0.17 | 4.12 | 1.65 | 5.77 |
| French Guiana | 0.08 | 0.00 | 0.08 | 0.14 | 0.00 | 0.14 |
| Guatemala | 0.05 | 0.03 | 0.08 | 1.02 | 5.27 | 6.30 |
| Guyana | 0.17 | 0.04 | 0.20 | 0.14 | 0.52 | 0.66 |
| Haiti | 0.03 | 0.00 | 0.03 | 8.58 | 0.02 | 8.60 |
| Honduras | 0.05 | 0.03 | 0.08 | 0.88 | 2.63 | 3.51 |
| Nicaragua | 0.09 | 0.03 | 0.11 | 1.57 | 2.12 | 3.70 |
| Panama | 0.02 | 0.00 | 0.02 | 0.90 | 0.00 | 0.90 |
| Peru | 0.50 | 0.06 | 0.57 | 3.87 | 1.70 | 5.57 |
| Suriname | 0.02 | 0.11 | 0.13 | 0.01 | 0.05 | 0.06 |
| Venezuela | 0.15 | 0.49 | 0.64 | 0.22 | 6.24 | 6.46 |
| **Total** | **6.06** | **2.17** | **8.23** | **41.13** | **50.23** | **91.37** |
|  |  |  |  |  |  |  |
| **EMRO** |  |  |  |  |  |  |
| Afghanistan | 0.05 | 0.21 | 0.26 | 4.56 | 12.53 | 17.10 |
| Djibouti | 0.00 | 0.02 | 0.02 | 0.02 | 0.41 | 0.43 |
| Iran | 0.01 | 0.26 | 0.27 | 0.15 | 2.72 | 2.87 |
| Pakistan | 0.21 | 0.55 | 0.76 | 30.74 | 68.30 | 99.04 |
| Saudi Arabia | 0.01 | 0.03 | 0.03 | 0.72 | 1.22 | 1.94 |
| Somalia | 0.58 | 0.06 | 0.64 | 10.04 | 0.55 | 10.59 |
| Sudan | 1.52 | 1.06 | 2.57 | 28.99 | 6.84 | 35.83 |
| Yemen | 0.21 | 0.25 | 0.47 | 15.93 | 5.72 | 21.65 |
| **Total** | **2.60** | **2.43** | **5.02** | **91.14** | **98.30** | **189.44** |
|  |  |  |  |  |  |  |
| **EURO** |  |  |  |  |  |  |
| Kyrgyzstan | 0.00 | 0.02 | 0.02 | 0.00 | 1.20 | 1.20 |
| Tajikistan | 0.00 | 0.02 | 0.02 | 0.00 | 2.16 | 2.16 |
| **Total** | **0.00** | **0.03** | **0.03** | **0.00** | **3.36** | **3.36** |
|  |  |  |  |  |  |  |
| **SEARO** |  |  |  |  |  |  |
| Bangladesh | 0.02 | 0.05 | 0.07 | 15.12 | 47.99 | 63.11 |
| Bhutan | 0.01 | 0.00 | 0.01 | 0.79 | 0.47 | 1.26 |
| India | 1.63 | 1.32 | 2.95 | 414.53 | 535.59 | 950.12 |
| Indonesia | 1.31 | 0.43 | 1.74 | 68.59 | 81.93 | 150.52 |
| Myanmar | 0.68 | 0.01 | 0.69 | 42.88 | 1.91 | 44.79 |
| Nepal | 0.01 | 0.02 | 0.04 | 3.40 | 6.15 | 9.54 |
| Sri Lanka | 0.01 | 0.03 | 0.05 | 1.75 | 7.53 | 9.28 |
| Thailand | 0.20 | 0.27 | 0.47 | 16.53 | 30.53 | 47.06 |
| Timor-Leste | 0.01 | 0.00 | 0.01 | 0.96 | 0.00 | 0.96 |
| **Total** | **3.89** | **2.14** | **6.03** | **564.54** | **712.08** | **1276.62** |
|  |  |  |  |  |  |  |
| **WPRO** |  |  |  |  |  |  |
| Cambodia | 0.18 | 0.01 | 0.19 | 10.77 | 2.55 | 13.33 |
| China | 0.20 | 0.16 | 0.37 | 17.13 | 20.32 | 37.45 |
| Lao People's Dem. Republic | 0.24 | 0.00 | 0.24 | 5.30 | 0.01 | 5.31 |
| Malaysia | 0.23 | 0.00 | 0.23 | 6.29 | 16.17 | 22.46 |
| Papua New Guinea | 0.40 | 0.00 | 0.40 | 4.11 | 0.00 | 4.11 |
| Philippines | 0.16 | 0.06 | 0.22 | 26.95 | 20.41 | 47.35 |
| Solomon Islands | 0.02 | 0.00 | 0.02 | 0.43 | 0.00 | 0.43 |
| Vanuatu | 0.01 | 0.00 | 0.01 | 0.22 | 0.00 | 0.22 |
| Viet Nam | 0.16 | 0.18 | 0.33 | 19.31 | 53.31 | 72.62 |
| **Total** | **1.60** | **0.41** | **2.02** | **90.51** | **112.77** | **203.28** |
|  |  |  |  |  |  |  |
| **Global Totals** | **29.85** | **10.29** | **40.14** | **1388.39** | **985.17** | **2373.56** |
